# Genome-wide identification and characterization of abiotic-stress responsive *SOD* (*superoxide dismutase*) gene family in *Brassica juncea* and *B. rapa*

**DOI:** 10.1186/s12864-019-5593-5

**Published:** 2019-03-19

**Authors:** Deepika Verma, Neha Lakhanpal, Kashmir Singh

**Affiliations:** Department of Biotechnology, BMS Block I, Panjab University, Sector 25, Panjab University, Chandigarh, 160014 India

**Keywords:** *Superoxide dismutase*, Gene family, *Brassica juncea*, *B. rapa*, Genome-wide analysis, Abiotic stress, Differential expression

## Abstract

**Background:**

Abiotic stresses like drought, heat, cold and salinity cause major productivity loss in the rapeseed-mustard crops (*Brassica*). Major efforts have been made in the past to identify genes that provide resistance against such stresses. Superoxide dismutase (SOD) proteins, member of the metallo-enzyme family play vital role in protecting plants against abiotic stresses. In the present study, genome-wide analysis of abiotic stress responsive *SOD* gene family has been done in *B. juncea* and *B. rapa*.

**Results:**

A total of 29 and 18 *SOD* genes were identified in *B. juncea* and *B. rapa* respectively and chromosome location mapping indicated their wide distribution across genome. On the basis of domain composition, the *SODs* were phylogenetically classified into sub-groups which was also substantiated by the gene structure and sub-cellular locations of SOD proteins. Functional annotation of *SODs* was also done by Gene Ontology (GO) mapping and the result was corroborated by the identified *cis*-regulatory elements in the promoter region of *SOD* genes. Based on FPKM analysis of SRA data available for drought, heat and salt stress, we identified 14 and 10 abiotic stress responsive *SOD* genes in *B. rapa* and *B. juncea* respectively*.* The differential expression analysis under drought and heat stress of identified abiotic-stress responsive *SOD* genes was done through quantitative Real Time PCR.

**Conclusion:**

We identified abiotic-stress responsive genes that could help in improving the plant tolerance against abiotic stresses. This was the first study to describe the genome-wide analysis of *SOD* gene family in *B. rapa* and *B. juncea*, and the results will help in laying basic ground for future work of cloning and functional validation of *SOD* genes during abiotic stresses leading to *Brassica* crop improvement.

**Electronic supplementary material:**

The online version of this article (10.1186/s12864-019-5593-5) contains supplementary material, which is available to authorized users.

## Background

The genus *Brassica* (*Brassicaceae* (*Cruiciferae*) family) comprises of species of important vegetable and oilseed crops. Six interrelated species share major portion of this family, amongst which three are diploids: *Brassica rapa* (AA 2n = 20, Chinese cabbage, turnip), *B. nigra* (BB 2n = 16, Black mustard), *B. oleracea* (CC 2n = 18, Cauliflower, broccoli), the other three species are allopolyploids formed by interspecies hybridization of the diploid species, namely, *B. napus* (AACC 2n = 38, oilseed rape or rapeseed), *B. juncea (*AABB 2n = 36, Indian or brown mustard) and *B. carinata* (BBCC 2n = 34, Ethiopian mustard) [1, 2]. These crops are mainly grown for oil, condiments, vegetables and fodder [3].

*B. rapa* is primarily grown as an oilseed producing crop in China, that shares ancestral history with the *Arabidopsis thaliana* and has experienced whole genome triplication event (WGT) 13–17 million years ago [4, 5]. The present day *B. rapa* is said to be evolved from the eight chromosomes carrying ancestral *Brassicaceae* karyotype (ABK). The chromosomes underwent differential fractionation in the gene content giving rise to the three genome fractions: least fractionated (LF), medium fractionated (MF1) and most fractionated (MF2) covering the entire 10 chromosomes of the present day *B rapa* [6]. The cultivation is best achieved at a pH range of 4.5–8.5 with well-drained moist soil [7].

*B. juncea* is a crucial agricultural and economical crop cultivated widely in India and China [8]. The regions, where *B. nigra* and *B. rapa* are cultivated extensively, also produced higher yields of *B. juncea* [9]. *B. juncea* is said to be evolved by many polyploidisation events giving rise to the present day species [10]*.* It is a major oilseed crop and also known for its medicinal properties. It is rich in Vitamin A, Vitamin C, calcium, potassium, iron, riboflavin, thiamine and β-carotene content. The production of this crop in India majorly lies in the northern and western part or 20°N of latitude and India is the largest producer of *Brassica* crops in Asia. The rapeseed-mustard crop production observed for the period 2015-2017according to FAO stats was more than 8 million tons [11].

Plants undergo various abiotic and biotic stresses at all times. The molecular action of each type of stress is different that eventually leads to plant death. Plants use different defensive mechanisms to fight against abiotic and biotic stress factors, like the production of defensive proteins and enzymes [12]. There have been reports on the effects of various abiotic and biotic stresses on *Brassica*: the effect of high temperature was observed to cause reduction in germination and seedling survival rate in the rapeseed-mustard crops [13]. Another study reported the decrease in the stem height of rapeseed cultivars due to water deficiency which ultimately led to the decline in photosynthetic activity [14]. The rapeseed-mustard production has decreased over the years owing to the abiotic stresses like drought and salinity [11].

Cellular metabolism produces reactive oxygen species (ROS) like superoxide ion (O^2−^), hydrogen peroxide (H_2_O_2_) and hydroxyl radicals (OH^−^) that leads to cell death. To deal with such ROS species, aerobic organisms have developed antioxidative defense mechanisms that comprise of antioxidants like ascorbate, glutathione, and guardian enzymes such as superoxide dismutases (SODs), catalase (CAT), and peroxidase (POX) [15].

SOD, member of the metalloenzyme family, propels the disproportion of O^2−^ ion into hydrogen peroxide (H_2_O_2_) and oxygen (O_2_) molecules. On the basis of metal cofactors, SODs can be categorized into 3 groups: copper/zinc SOD (CSD), iron SOD (FSD) and manganese SOD (MSD). Encoded by the nuclear genes, SOD proteins are located in various compartments of the cell, wherein CSD is localized in the mitochondria, chloroplast and cytosol. MSD is believed to be mainly located in the mitochondria and also in peroxisomes, whereas FSD occurs in mitochondria, chloroplast and peroxisomes [16]. High sequence similarity was observed in MSD and FSD, whereas CSD is distinct. There are evidences that animals & fungi contain CSD and MSD, whereas plants & bacteria have all three forms of SOD [17].

*SOD* family and its response to stress has been well studied and reported previously in plants like *Zea mays, Arabidopsis thaliana, Medicago sativa, Musa acuminata, Triticum aestivum, Gossypium hirsutum* and various other known plants, wherein the gene number varies from 7 to a maximum of 23 with *A. thaliana* and *Z. mays* containing 7 [17], *G. hirsutum* with 18 [16] and *T. aestivum* having 23 genes [18]. SOD has been described to be involved in various abiotic stress related responses such as heat, drought, salt, low temperature conditions [16, 18, 19]. In *B. napus* high SOD activity has been observed in salt-tolerant varieties compared to salt-sensitive one. [20]. Another study reported the improved resistance towards drought stress in the transgenic rice transformed with *MnSOD* gene from pea plant [21]. Transgenic *Cassava* lines over-producing *Cu-ZnSOD* were tested against spider mite, *Tetranychus cinnabarinus*, and these lines showed less damage as compared to the wild type [22]. Transgenic maize lines incorporated with *FeSOD* gene from *A. thaliana* showed improved tolerance towards methyl viologen (a potent oxidative stress inducer) and had increased growth rate [23].

The physiological importance of *SOD* family and its potential applicability in improving the efficacy of plant tolerance towards stress encouraged the genome wide identification and characterization of these genes in *B. rapa* and *B. juncea*. The classification and characterization of the identified genes was done based on structural diversification of the SOD proteins and their distribution across genome. Also the abiotic-stress responsive *SODs* were identified in both *B. rapa* and *B. juncea.*

## Methods

### Identification of SOD genes in *B. juncea* and *B. rapa*

The identification of SOD genes was carried out in two steps. First, the proteome of *B. juncea* and *B. rapa* was downloaded from *Brassica* database (BRAD) (http://brassicadb.org/brad/) and a local database was constructed for Stand Alone Blast. Next, the reported amino acid sequences of SOD proteins from six related plant species *B. napus, B. oleracea*, *A. thaliana, A. lyrata, Raphanus sativus, Camelina sativa* were retrieved using the information available on National Center for Biotechnology Information (NCBI) protein database (https://www.ncbi.nlm.nih.gov/protein) and was used as a query file against the above constructed local *Brassica* protein database.

Second, Hidden Markov Model (HMM) profiles of Cu-ZnSOD (PF00080) and Fe-MnSOD (PF02777 and PF00081) were downloaded from the pfam database (https://pfam.xfam.org/) and the above identified protein sequences were searched against these HMM profiles using an e-value cut-off of 1e-05. The candidate sequences obtained were considered as *SOD* genes for *B. juncea* and *B. rapa.* The protein sequences were further tested for the presence of SOD domains (pfam00080, pfam02777 and pfam00081) at various servers like InterProScan (https://www.ebi.ac.uk/interpro/search/sequence-search), SMART (http://smart.embl-heidelberg.de/), ScanProsite (https://prosite.expasy.org/scanprosite/), and NCBI Conserved Domain Search (https://www.ncbi.nlm.nih.gov/Structure/cdd/wrpsb.cgi) [24–27].

The computation of Physical and chemical properties of SOD proteins was done using the ProtParam tool (https://web.expasy.org/protparam/) and parameters like molecular weight, theoretical isoelectric point (pI) and amino acid composition were predicted [28].

### Chromosomal distribution of SOD genes

Information about the *SOD* genes and their chromosomal locations were collected from Brassica Database and NCBI. The *B. rapa SODs* were searched using the nr (non-redundant) database and the hit with maximum identity was used to locate the gene position. The position was also verified by the Plant Ensembl portal (https://plants.ensembl.org/Brassica_rapa). For the chromosomal mapping of *B. juncea SOD* genes, the information available on BRAD was used.

### Subcellular localization, conserved motifs and gene structure analysis

Subcellular location of SOD proteins was predicted using ProtComp version 9.0 server (http://www.softberry.com). Search for conserved motifs of SOD proteins was done using the Multiple Expectation Maximization for Motif Elicitation (MEME- Suite version 5.0.1) (http://meme-suite.org/) using the default settings, except the number of motif was set to 10 and minimum and maximum motif widths were changed to 20 and 150 respectively [29]. The visualization of exon-intron positions was done through an online software Gene Structure Display Server 2.0 (http://gsds.cbi.pku.edu.cn/) [30].

### Functional prediction of SOD genes

The annotation of gene function was carried out through Blast2GO tool (https://www.blast2go.com/) [31]. The analysis was based mainly on three aspects: biological processes, molecular functions and cellular components.

### Phylogenetic analysis

To investigate the phylogenetic relationship between *B. juncea* and *B. rapa*, full length coding sequences of the genes were aligned using Multiple Alignment using Fast Fourier Transform (MAFFT) (https://www.ebi.ac.uk/Tools/msa/mafft/) [32], Clustal omega (https://www.ebi.ac.uk/Tools/msa/clustalo/) [33] and Multiple Sequence Comparison by Log-Expectation (MUSCLE) (https://www.ebi.ac.uk/Tools/msa/muscle/). Sequence alignment was carried out for the protein SOD sequences using Clustal W program and the phylogenetic tree was constructed using Mega 7 software (https://www.megasoftware.net/) using the Neighbor-Joining method tested by 1000 Bootstrap replicates. The evolutionary distances were computed using Poisson correction method. For the evolutionary relationship study between *B. juncea* SODs and orthologous species, SOD protein sequences of 6 plant species (*A. thaliana, B. napus, B. rapa, C. sativa, R. sativus* and *Z. mays*) were downloaded from NCBI. A total of 80 protein sequences were used for orthologous tree construction. A similar approach was used for the construction of phylogenetic tree of *B. rapa*, wherein SODs of *B. rapa* and *A. thaliana* were aligned and the alignment result was further used for the tree generation.

### Analysis of cis-regulatory elements

For the identification of *cis*-regulatory elements in *SOD* genes, 1500 bp region upstream of the translation start site was retrieved by aligning the coding sequences with the genomic sequences. Regulatory elements were then predicted using the PlantCARE database (http://bioinformatics.psb.ugent.be/webtools/plantcare/html/) [34].

### Identification of abiotic stress-responsive SOD genes

In order to accomplish the task of identifying abiotic stress-responsive *SOD* genes, fasta file of all the identified *SOD* coding sequences was prepared. Next, the high-throughput RNA-seq data of CS52 and varuna variety of *B. juncea* were retrieved from Sequence Read Archive (SRA) database with the project number SRP055678, SRP051212 and SRP063855 for salt, drought, heat and cold stresses [35–37] respectively using NCBI SRA server (http://www.ncbi.nlm.nih.gov/sra). The drought stress data was available for 3 h and 12 h time points, whereas the heat stress data was for 30 min and 4 h time points. Salt stress data was provided for 24 h. The cold stressed samples were taken at different developmental stages (5,10,15,20,25,30 Days After Pollination) for 6 h and 12 h time points.

Similarly, RNA-seq data of *B. rapa* non-heading Chinese cabbage (heat-sensitive and heat-tolerant) and (drought-tolerant and drought-sensitive) was retrieved with the project number SRP064703 and SRP064814 for heat and drought stresses [38, 39] respectively. The expression level of all *SOD* genes were assessed by RSEM (RNA-Seq by Expectation Maximization) as fragments per kilobase of transcript per million fragments mapped (FPKM) using Trinity-V 2.03 [40]. EdgeR was used to calculate the differential gene expression of all *SOD* genes using a cut-off of four fold change in gene expression and *p* value of 0.05. Heat maps were generated using Hierarchical Clustering Explorer 3.5 (http://www.cs.umd.edu/hcil/hce/). Based on the Heat maps generated, the differentially expressed genes were selected and further used for validation through qRT-PCR.

### Plant growth conditions, treatments and sampling

*B. juncea* and *B. rapa* seeds were sterilized and grown in pots containing soil:soil rite in the ratio 2:1 at an optimum temperature of 20–24 °C with 16/8 h (light/dark) condition in the Plant growth chamber at the Department of Biotechnology, Panjab University, Chandigarh. Drought and Heat stress treatment was given to both the *B. juncea* and *B. rapa* plants in triplicates. Drought-stress was imposed by two means; (i) on 20 days old plants by using 20% (*w*/*v*) PEG 8000 solution for 4 h and 8 h and (ii) on 30 days old plants by with-holding water for 7 days. After completion of stress period, leaves were excised and snap frozen in Liquid N_2_. The soil moisture contents (wt/wt) were measured in plant where drought stress was given by withholding water. In order to provide heat stress, 20 days old plants were placed at 40 °C in growth chamber for 4 h and 8 h and the control were placed at 20 °C. Leaf samples were snap frozen and preserved at − 80 °C till further use. All the experiments were performed in triplicates with three biological replicates along with their control samples.

### RNA isolation, cDNA synthesis & qPCR analysis

Total RNA was isolated from the frozen leaf samples of drought and heat stressed *B. juncea* and *B. rapa* plants [41].cDNA was prepared using Superscript III first strand cDNA synthesis kit (Invitrogen USA). Tonoplastic Intrinsic Proteins-41 (TIPS-41) and Actin-7 (ACT-7) were the two housekeeping genes used as internal control for normalization of qPCR results of *B. juncea* and *B. rapa* [42]. Primer 3 software (http://primer3.ut.ee/) was used to design real time primers (Additional file 1). qPCR validation was done using three biological replicates of control and stressed samples with the aid of Bio-Rad CFX96 Real-Time PCR detection system. The conditions followed for the experiment were 95 °C for 7 min, followed by 40 cycles of 95 °C for 20 s, Tm for 20 s and 72 °C for 20 s. Tm was standardized for all the genes using semi-quantitative PCR. The calculation of expression fold change was done using the 2(−∆∆C_T_) method [43].

## Results

### Identification, genome wide distribution and chromosomal mapping of SOD genes in Brassica species

With the aid of published genome*,* the task of identifying *SOD* genes in *B. juncea* and *B. rapa* was achieved. The putative hits identified after protein sequence analysis with reported SODs were examined for the presence of SOD domains using different bioinformatics tools like CDD, InterProScan, Pfam, SMART and ScanProsite. Finally, 29 and 18 candidate *SOD* genes were obtained in *B. juncea* and *B. rapa* respectively*.* We propose that these *SOD* genes be named *CSD, MSD* and FSD. The gene names, sequence IDs and genomic positions of identified *SODs* are mentioned in Tables 1 and 2. The *SOD* gene number in case of *B. juncea* was higher as compared to 7 in *A. thaliana,* 18 in *G. hirsutum,* 8 in *S. bicolor,* 23 in *T. aestivum,* 9 in *S. lycopersicum* [16–19, 44].

**Table 1:** Identification of *SOD* genes from *B. juncea* and their physico-chemical and bio-chemical properties

Gene Name	Sequence ID	Chromosome	Genomic position	Intron number	Length (aa)	MW (kDA)	pI	Predicted pfam domain	Subcellular prediction by PC
BjuACSD1a	BjuA044056	A09	A09:53931004..53933846 (+)	6	260	27.13	6.29	CZ,RVT_3	Cytoplasm
BjuACSD1b	BjuA019526	A09	A09:54112953..54114117 (−)	6	152	15.18	5.64	CZ	Cytoplasm
BjuBCSD1c	BjuB029125	B04	B04:19092172..19095235 (−)	7	335	35.91	8.53	CZ, R-L18p	Cytoplasm
BjuBCSD1d	BjuB004106	–	–		260	27.13	6.29	CZ, RVT_3	Cytoplasm
BjuACSD2a	BjuA016336	A04	A04:17609587..17610902 (+)	7	208	21.46	6.54	CZ	Chloroplast
BjuACSD2b	BjuA000975	A07	A07:20085852..20088762 (+)	8	399	43.42	5.04	CZ, HLH	Nucleus
BjuACSD3	BjuA039051	A10	A10:14039402..14040884 (−)	3	108	11.21	6.11	CZ	Peroxisome
BjuACSD4	BjuA031027	A08	A08:24172096..24173536 (+)	5	317	33.75	6.35	CZ, HMA	Cytoplasm
BjuACSD5	BjuA033232	A09	A09:10507338..10508161 (−)	2	219	23.62	6.82	CZ	Cytoplasm
BjuACSD6	BjuA036999	A09	A09:52756456..52757949 (+)	5	319	33.65	5.41	CZ, HMA	Cytoplasm
BjuBCSD7	BjuB048313	B02	B02:34694766..34697823 (+)	9	538	58.42	5.72	CZ, HMA	Chloroplast
BjuBCSD8	BjuB033224	B03	B03:2138821..2140379 (−)	4	308	32.62	6.21	CZ, HMA	Cytoplasm
BjuAMSD1a	BjuA033927	A01	A01:37574252..37575325 (−)	4	163	17.92	7.01	IMA, IMC	Mitochondrion
BjuAMSD1b	BjuA020544	A05	A05:28269085..28270372 (+)	5	231	25.41	8.47	IMA, IMC	Mitochondrion
BjuAMSD1c	BjuA035793	A09	A09:45173051..45179490 (−)	3	310	34.52	9.57	IMA, IMC, R-S26e	Mitochondrion
BjuAMSD1d	BjuA037317	A10	A10:711262..722525 (−)	7	323	35.03	5.02	IMC, GSHPx, Gar1	None
BjuBMSD1e	BjuB024110	B01	B01:22343773..22344375 (−)	1	84	8.79	6.55	–	Mitochondrion
BjuBMSD1f	BjuB023608	B01	B01:42834958..42836295 (−)	4	161	17.65	6.36	IMA, IMC	Mitochondrion
BjuBMSD1g	BjuB006884	B07	B07:3015680..3017012 (+)	5	231	25.51	8.46	IMA, IMC	Mitochondrion
BjuAFSD1a	BjuA004236	A01	A01:9464296..9465978 (−)	6	212	23.84	5.97	IMA, IMC	Chloroplast
BjuBFSD1b	BjuB014294	–	–		646	70.89	5.84	IMA, IMC	none
BjuAFSD2a	BjuA010212	A03	A03:8427338..8429115 (+)	8	295	33.86	4.91	IMA, IMC	Chloroplast
BjuBFSD2b	BjuB035345	B02	B02:19080775..19081998 (−)	3	174	19.78	4.36	IMC, GSHPx, Gar1	Chloroplast
BjuBFSD2c	BjuB015833	B08	B08:16094351..16096146 (+)	8	298	33.79	4.86	IMA, IMC	Chloroplast
BjuAFSD3a	BjuA023484	A06	A06:20407619..20409162 (+)	7	233	26.77	5.98	IMA, IMC	Chloroplast
BjuAFSD3b	BjuA032504	A09	A09:5297572..5299258 (+)	6	233	26.78	5.88	IMA, IMC	Chloroplast
BjuAFSD3c	BjuA032067	–			258	29.72	8.39	IMA, IMC	Chloroplast
BjuAFSD3d	BjuA023518	–			240	27.7	6.45	IMA, IMC	Chloroplast
BjuBFSD3e	BjuB044804	B02	B02:60867392..60868959 (+)	5	240	27.69	6.45	IMA, IMC	Chloroplast

**Table 2:** Identification of *SOD* genes from *B. rapa* and their physico-chemical and bio-chemical properties

Gene Name	Sequence ID	Genomic Location	Intron number	Length (aa)	MW (kDA)	pI	Predicted pfam domain	Subcellular prediction by PC
BraCSD1a	BraA06001242	A6:3058529..3060316	2	394	42,496.33	8.97	CZ, R_L18p	Cytoplasm
BraCSD1b	BraA09006623	A9:345460824..35462750	6	152	15,179.77	5.64	CZ	Cytoplasm
BraCSD2a	BraA04002032	A4:12324482..12326015	7	207	21,368.08	6.54	CZ	Cytoplasm
BraCSD2b	BraA07001861			514	55,316.81	5.69	CZ, HLH	Nucleus
BraCSD3	BraA10001953	A10:11324155..11325820	5	153	15,793.54	6.74	CZ	Peroxisome
BraCSD4	BraA08003518	A8:19894341..19895990	5	316	33,636.2	6.35	CZ, HMA	Cytoplasm
BraCSD5	BraA09001362	A9:5914443..5920054	2	219	23,591.78	5.76	CZ	Cytoplasm
BraCSD6	BraA09006425	A9:34559418..34561060	5	502	54,532.3	5.32	CZ, HMA	Chloroplast
BraMSD1a	BraA01004312	A1:27214694..27216183	5	231	25,455.09	8.77	IMA, IMC	Mitochondrion
BraMSD1b	BraA05004003	A5:21906786..21908342	5	231	25,413.91	8.47	IMA, IMC	Mitochondrion
BraMSD1c	BraA09004996	A9:28178287..28179789	5	241	26,970.71	6.21	IMA, IMC	Mitochondrion
BraMSD2	BraA10000617			230	24,929.58	5.03	IMC, Gar-1	None
BraFSD1	Scaffold000083.115			657	72,175.59	5.69	IMA, IMC, zf-LSD1, Peptidase_C14	None
BraFSD2a	Scaffold000070.295	A3:6771694..6773783	7	328	37,654.66	4.67	IMA, IMC	Chloroplast
BraFSD2b	BraSca000188	A4:12536379..12538015	3	120	13,831.98	4.2	IMC	Chloroplast
BraFSD3a	BraA09000711	A1:8963410..8965500	7	263	30,169.28	7.75	IMA, IMC	Chloroplast
BraFSD3b	Scaffold000004.555	A6:16473983..16475790	7	227	26,020.46	8.4	IMA, IMC	Chloroplast
BraFSD4	BraA03001646	A3:6771694..6773783	0	54	6200.48	3.76	None	Chloroplast

**Fig. 1:**
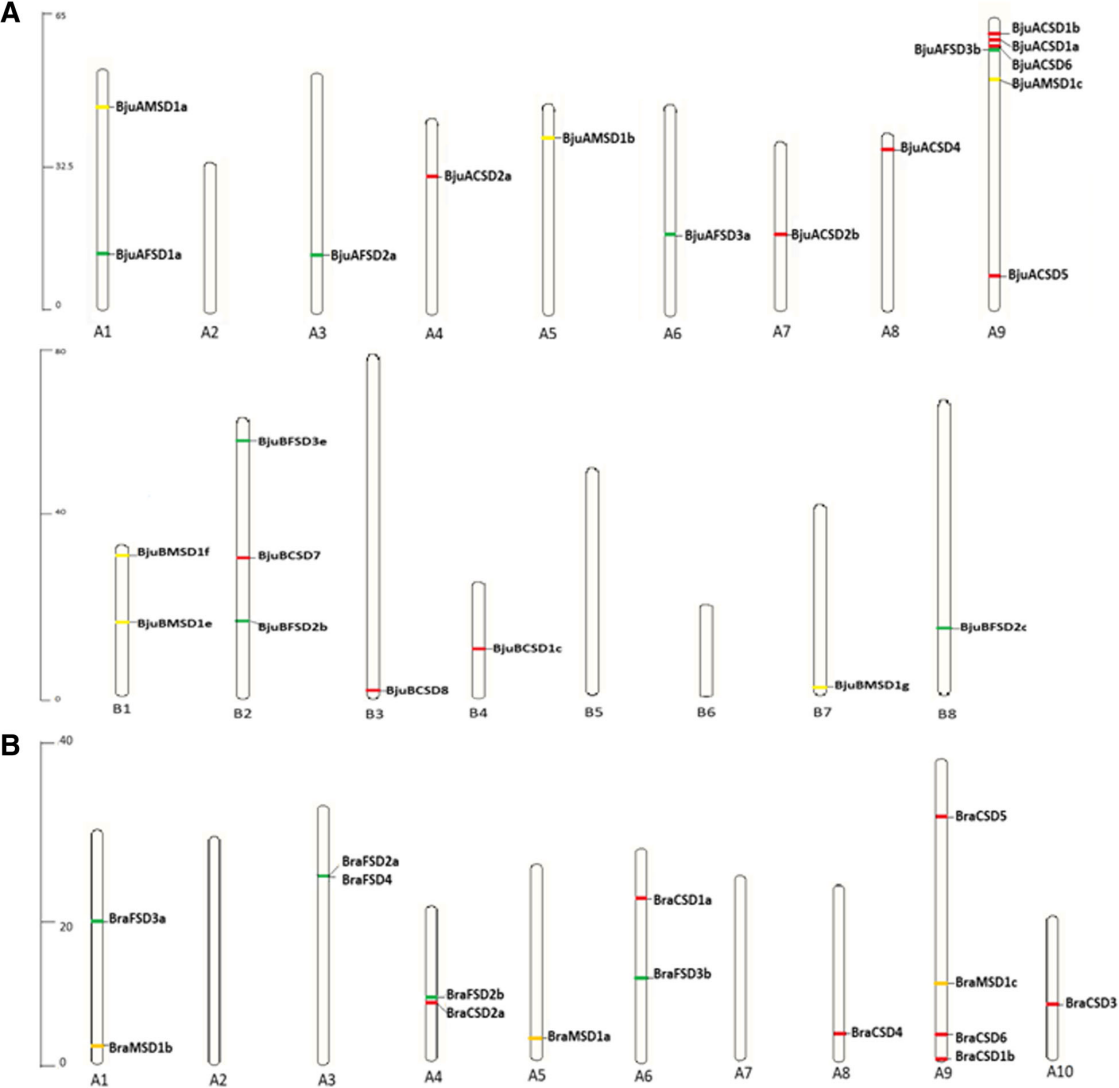
Chromosomal mapping of *SOD* genes (**a**) on A and B chromosomes of *Brassica juncea* and (**b**) A chromosomes of *B. rapa*. The black lines indicate the chromosomal position of respective genes. The chromosome numbers are represented below the chromosomes and vertical scale is showing the size of chromosome, wherein *CSDs, MSDs* and *FSDs* are marked with red, yellow and green colors respectively

The *SOD* genes identified in *B.juncea* were present in both A and B sub-genomes wherein, majority of *BjuSODs* were located on chromosome A9 (6 genes) followed by chromosome B2 (3 genes), chromosome A1, A10, B3 and B1 had 2 genes on each chromosome, A4, B4, A8, A7, B7, A5, A6, B8 and A3 each had one gene and the map showing the chromosomal location was constructed (Fig. 1a). Arrangement of *SOD* genes on *B. rapa* chromosomes was also predicted. Chromosome A9 of *B. rapa* had 4 genes, while chromosome A1, 3, 4 and 6 each carried 2 *SOD* genes as shown in the chromosomal map (Fig. 1b). Our results are suggestive of the fact that the *SOD* genes are widely distributed throughout the genomes of *B. rapa* and *B. juncea.*

### Structural investigation of SOD genes

**Fig. 2:**
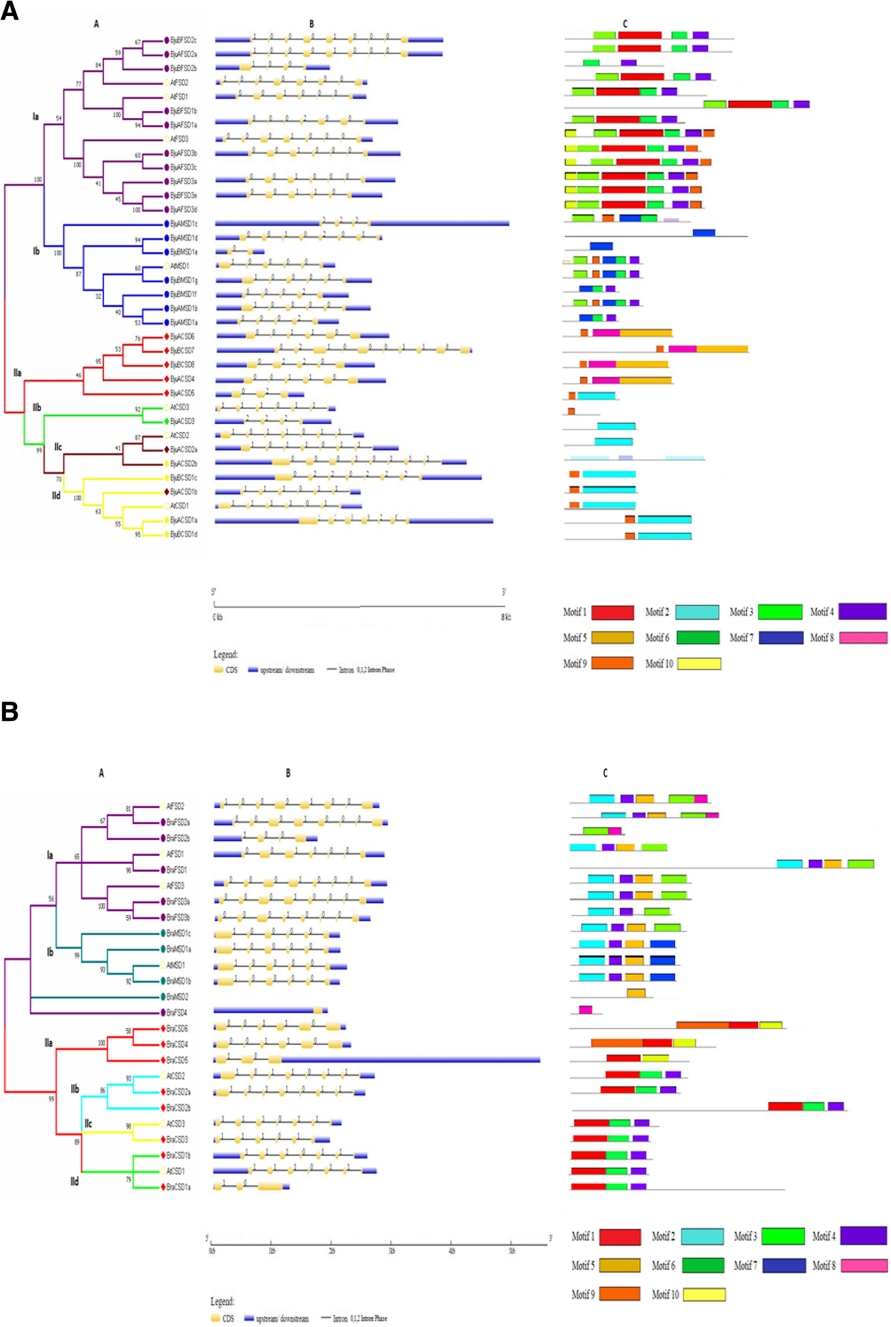
**a** Phyolgenetic tree, Gene structure and motif analysis of *BjSOD* genes. The evolutionary relationship between *B. juncea* and *A. thaliana* is shown in (A). The intron/exon organization in *SOD* genes is represented; yellow boxes depicting exons separated by introns with thin lines. Blue boxes indicated UTRs in (B), 0 = Intron phase 0, 1 = Intron phase 1 and 2 = Intron phase 2. Conserved motif analysis was done using MEME motif based sequence analysis tool and the conserved motifs are shown (C). In total, 10 conserved domains were identified. **b** Phyolgenetic tree, Gene structure and motif analysis of *BraSOD* genes. The evolutionary relationship between *B. rapa* and *A. thaliana* is shown in (A). The intron/exon organization in *SOD* genes is represented; yellow boxes depicting exons separated by introns with thin lines. Blue boxes indicated UTRs in (B), 0 = Intron phase 0, 1 = Intron phase 1 and 2 = Intron phase 2. Conserved motif analysis was done using MEME motif based sequence analysis tool and the conserved motifs are shown (C). In total, 10 conserved domains were identified

The structural evolution of *SOD* genes was observed through the intron-exon structures generated by the GSDS server as depicted in Fig. 2a-b. The intron number varied from 2 to 9, 1–7 and 3–8 in *BjuCSDs, BjuMSDs* and *BjuFSDs* respectively (Table 1). In case of *B. rapa* the intron number ranged from 2 to 7, 5 and 3–7 in *BraCSD, BraMSD* and *BraFSD* respectively (Table 2). The intron occurred in three phases: 0 phase, 1 phase and 2 phase. Majority of the genes of *B. juncea* had 0 phase introns with a percentage of about 58.19%, whereas 31.64% introns were in 1 phase and only 10.17% intron appeared in phase 2. The introns in case of *B. rapa* were observed to occur in 0 phase and 1 phase only, wherein 61.97% were in 0 phase and 38.03% in 1 phase. The introns exhibited ‘AG’ and ‘GT’ nucleotides at the 5′ and 3′ splice sites respectively. Similar intron-exon organization was also observed in *A. thaliana* also.

### Physico-chemical characterization of SOD proteins

The predicted protein encoded by *SOD* genes and their physiochemical analysis revealed the range of below mentioned parameters like: amino acid length 84–646 (minimum-maximum), molecular weights 8.79–70.88 kDa, and pI 4.36–9.57 in case of *B. juncea.* The obtained variation in case of *B. rapa* was 54–657, 6.20–72.17 kDa and 3.76–8.97 for amino acid length, molecular weight and pI respectively (Tables 1 and 2). The SOD proteins were classified on the basis of their domain composition into 3 groups: 12 BjuCSD, 7 BjuMSD and 10 BjuFSD. The classification in *B. rapa* was similar with 8 BraCSD, 4 BraMSD and 6 BraFSD. The analysis of putative SOD protein sequences using Pfam database predicted the presence of Cu-ZnSOD domain (pfam00080) in all Cu-ZnSODs, whereas Fe-MnSOD alpha-hairpin domain and Fe-MnSOD C-terminal domain (pfam 00081, pfam02777 respectively) were present in the Fe-MnSODs.

Based on the pI prediction, the BjuCSDs and BjuFSDs were acidic in nature with the exception of BjuBCSD1c and BjuAFSD3c which were basic in nature*.* The BjuMSDs were of mixed nature. Similarly, BraCSDs and BraFSDs were acidic in nature with the exception of two being basic in nature i.e. BraCSD1a and BraFSD3b, whereas again the BraMSDs were mixed in nature.

The sub-cellular localization of the SOD proteins predicted by ProtComp v9.0 showed that 8 members (BjuCSD 1a, 1b, 1c, 1d, 4, 5, 6, 8) were localized in the cytoplasm; 2 members (BjuCSD 2a and 7) were found in the chloroplastic region; BjuCSD 3 and 2b in the peroxisome and nucleus respectively. All BjuMSDs were likely to be located in the mitochondrion and BjuFSDs in the chloroplastic region. Likewise, for *B. rapa* the location of SOD proteins was anticipated as BraCSD 5,4,2a,1b,1a in the cytoplasm, BraCSD6 in chloroplast; BraCSD2b and 3 in the nucleus and peroxisome respectively. The location provided for the BraMSDs was mitochondrion and for BraFSDs was chloroplast.

### Protein structure analysis

The protein structure was predicted including its primary, secondary and tertiary structures. Also various metal binding sites, disulphide bond forming conserved residues, shell forming residues were predicted based on the tertiary structures obtained through I-TASSER. The SOD proteins were observed to exhibit high conservation pattern based on the multiple sequence alignment results. Many metal binding sites were found in CSDs, FSDs and MSDs Major sites found were copper atom binding sites (His-100, 102 and 174) and zinc binding sites (His-125,134 and Asp-137) in CSD proteins (Additional file 2a and Additional file 3a). Also a bridging histidine residue (His-117) and conserved cysteine residues (Cys-111 and 200) involved in disulphide bond formation. These residues are highly conserved in CSD proteins [45]. The metal binding conserved residues in MSD were His-55, 103 and 196 and Asp-192 [46] (Additional file 2b and Additional file 3b). In FSD proteins the conserved residue groups were His-44, 97 and 185 and Asp-181 forming a trigonal bypyramidal arrangement around the Fe atom. Fe atom is embedded in a cavity covered and protected by the shell forming residues His-47, Tyr-51, Gln-91 and Trp-197 [47] (Additional file 2c and Additional file 3c). All these residues are highly conserved in nature suggested by various reports.

The tertiary structures (Additional files 4, 5, and 6) and multiple sequence alignment results showed the difference between CSDs, FSDs and MSDs. CSD proteins showed eight anti-parallel β-strands. On the other hand FSD and MSD comprised of α-helices and anti-parallel β-sheets. FSDs had nine α-helices and three anti parallel β-sheets, whereas MSDs showed seven α-helices and three anti parallel β-strands. Additionally the conserved signature Cu^2+^ and Zn^2+^ − binding sites and metal binding site in FSD and MSD were also shown in the multiple sequence alignment of the proteins .

The domain structure for each SOD protein was analyzed using pfam which indicated the presence of ~ 140 aa residue (Cu-ZnSOD pfam00080) domain in all the CSD proteins in both *B. juncea* and *B. rapa.* Moreover, additional domains like heavy metal associated domain (HMA pfam00043) of ~ 57 aa residue was found in four BjuCSD4, 6, 7 and 8, BraCSD6 and BraCSD4; reverse transcriptase 3 (RVT-3 pfam13456) of ~ 66 aa residue was found in BjuACSD4 and BjuCSD1d; ribosomal_L18p (R_L18p pfam 00861) of ~ 118 aa residue was found in BjuBCSD1c and BraCSD1a; helix-loop helix (HLH pfam 00010) ~ 49 aa residue was found in BjuACSD2b and BraCSD2b*.*

In case of MSD, both the Iron/manganese superoxide dismutases, alpha-hairpin domain, pfam 00081 of ~ 85 and Iron/manganese superoxide dismutases, C-terminal domain, pfam 02777 of ~ 104 aa residue was detected in all the BjuMSD and BraMSD*.* Associated with the MSD was found three more domains namely Ribosomal_S26e (R_S26e pfam 01283) of ~ 107 aa residue in BjuAMSD1c; Gar1/Maf1 RNA binding region (Gar1 pfam 04410) of ~ 81 aa residue in BjuAMSD1d and BraMSD2; Glutathione peroxidase (GSHPx pfam 00255) of ~ 37 aa residue in BjuAMSD1d*.* The FSD proteins in both species showed the presence of both pfam 00081 and pfam 02777 domains of ~ 86 and 118 aa residue sequence respectively along with four extra domains namely pfam 00255 domain and pfam 04410 domain in BjuBFSD2b; Peptidase_C14 (P_C14 pfam 00656) domain of ~ 193 aa residue and LSD1 zinc finger (Zf-LSD1 pfam 06943) domain of ~ 23 aa residue in BraFSD1.

### Conserved motif analysis

The protein sequences of the predicted *SODs* were tested for the presence of the conserved motifs. The MEME server predicted a total of 10 conserved motifs widely distributed across the *SOD* sequences in both *B. juncea and B. rapa*. The motif sequences and domain patterns of the SOD proteins in both *B. juncea* and *B. rapa* are shown in Additional file 7a, b and Additional file 8a, b respectively .

Majority of BjuCSDs showed the presence of motifs 2 and 5 corresponding to AtCSDs (Fig. 2a). Pfam analysis of these motifs revealed their relatedness with the (Cu-ZnSOD domain pfam00080) which contains the CSD signatures and conserved Cu^2+^ and Zn^2+^ − binding sites (Additional file 7a, b). These motif signatures were further confirmed by ScanProsite which gave us similar results. The motif 8 was specifically related to BjuCSD 4, 8, 6 and 7*,* which corresponds with the HMA_2 (pfam00403) domain, seventy amino acid residues long that is associated with protein which detoxify metal ions. The BjuFSDs comprised of a combination of motifs 1,3,4,6, 9 and 10 which were related with the (Fe-MnSOD domain pfam00081, pfam02777), whereas BjuMSDs consisted of 3,4,6,7 and 9 motif number which included the Fe-MnSOD domain (pfam00081, pfam02777). Motif 1 and 10 was found to be associated with the BjuFSDs while motif 7 was associated with BjuMSDs*.* Motif 4 found in both FSD and MSD included the conserved metal-binding domain “DVWEHAYY” of FSD and MSD. The data was analyzed using pfam database and cross-checked with ScanProsite which supported our results.

A total of 10 motifs were identified for *B. rapa* shown in Fig. 2b. Motifs 1, 4, 6 and 10 were found in BraCSDs similar to the model plant AtCSDs. These motifs were associated with the (Cu-ZnSOD domain pfam00080), containing the conserved signature sequence and Cu^2+^ and Zn^2+^ − binding sites (Additional file 7b). Motif 9 was the additional motif found in three CSD (BraCSD 5, 6 and 4) which contained the HMA domain (pfam00403). The motifs identified in BraFSDs was a combination of 2, 3, 4, 5 and 8 which contained the (Fe-MnSOD domain pfam02777 and pfam00081) similar to all the AtFSDs. The BraMSDs contained the motif combination 2, 4, 5 and 7 which included the Fe-MnSOD domain (pfam00081 and pfam02777). The differentiating motifs between BraFSD and BraMSD were 8 and 7 respectively. Motif 3 contained the conserved metal-binding domain “DVWEHAYY” for FSD and MSD.

### GO analysis of SOD genes

The three characteristics of genes i.e. ‘Biological processes’, ‘molecular functions’, and ‘cellular components’ help us in understanding the functioning of proteins at the molecular level. The annotations of *SOD* genes of both *B. juncea* and *B. rapa* were predicted using Blast2GO tool. The ‘Cellular components’ annotation predicted the *SODs* to be present in cytosol, chloroplast, peroxisome, mitochondrion which matches the sub-cellular prediction results for the SOD proteins. According to the ‘Molecular functions’ characteristic the genes were involved in “superoxide dismutase activity” (GO:0004784), the *Cu-ZnSOD* genes had “copper ion binding” (GO:0005507) and “zinc ion binding” (GO:0008270), all the *Mn-FeSOD* gens had “metal ion binding” (GO:0046872). In addition, few *Cu-ZnSODs* presented the “superoxide dismutase copper chaperone activity” (GO:0016532). The annotation results for ‘Biological processes’ indicated that the genes possessed “removal of superoxide radicals” (GO:0019430) and “oxidation-reduction process” (GO:0055114). Also the *SOD* genes were found to be involved in response to biotic and abiotic stimulus, like bacterium (GO:0042742), light (GO:0071484), UV-B (GO:0071493), salt (GO:0009651) and ozone (GO:0010193). Gene ontology annotations prediction of the *SOD* genes in *B. juncea* and *B. rapa* was done considering the homology with *A. thaliana SODs* (Additional file 9a, b).

### Phylogenetic analysis

The evolutionary relatedness of *SODs* between *Brassica* and *A. thaliana* was explored by aligning multiple SOD protein sequences and constructing a phylogenetic tree for the identified *SOD* genes using CLUSTALW and MEGA 7.0 respectively. The *BjuSOD* genes were clustered into 2 main groups: CSD and FSD-MSD which were further divided into 6 sub-groups. Group I contained 2 sub-groups Ia, Ib represented by purple and blue colors respectively, where in each color represents the FSD and MSD; whereas Group II contained 4 sub-groups IIa, IIb, IIc and IId represented by red, green, brown and yellow colors respectively (Fig. 2a). Following the sub-cellular predictions (Table 1), the ordering of all the sub-groups of group I may be based on the sub-cellular locations of FSD and MSD as observed in SODs in *T. aestivum* [18]. The highly similar *BjuCSDs* were placed together in the phylogenetic tree. The peroxisomal *BjuACSD3,5* was clustered with the peroxisomal *AtCSD3* in sub-group IIb with a bootsrap value of (89%). The members of sub-group IIa clustered together the cytosolic *BjuCSD* with a bootsrap value of (53%) with the exception of *BjuBCSD7* which was chloroplastic in origin. Group IId members were predicted to be cytoplasmic in nature and were clustered together. Group IIc members were chloroplastic in origin and were placed together with a bootstrap value as high as (90%), but the nuclear *BjuACSD2b* was also placed in the same group. The results were in accordance with the previous proven studies with a few exceptions in case of *BjuCSD* arrangements. The tree showing *SOD* genes in *B. rapa* (Fig. 2b) was divided into 2 groups like *B. juncea*, which were further divided into sub-groups represented by different colors. Group I contained 2 sub-groups Ia and Ib represented by purple and dark green colors respectively, wherein purple shows FSD and dark green shows MSD. Group II contains 4 sub-groups, wherein different members of CSD are highlighted in red, sky blue, yellow and parrot green colors representing the sub-groups IIa, IIb, IIc and IId respectively. Two genes *BraMSD2* and *BraFSD4* were separately placed on the tree in group I.

Another phylogenetic tree constructed between *B. rapa* and *B. juncea* showed the clear clustering of most related *SODs* (Additional file 10). The clustering was clearly on the basis of sub- cellular localization prediction. For example, *BjuACSD5* and *BraCSD5*; *BjACSD2b* and *BraCSD2b*; *BjuACSD3* and *BraCSD3* were clustered together in same clades with very high bootstrap values (99, 98 and 98% respectively). On analysis, these genes were found to be located on same chromosomes as their counterparts, also they had similar intron number.

**Fig. 3:**
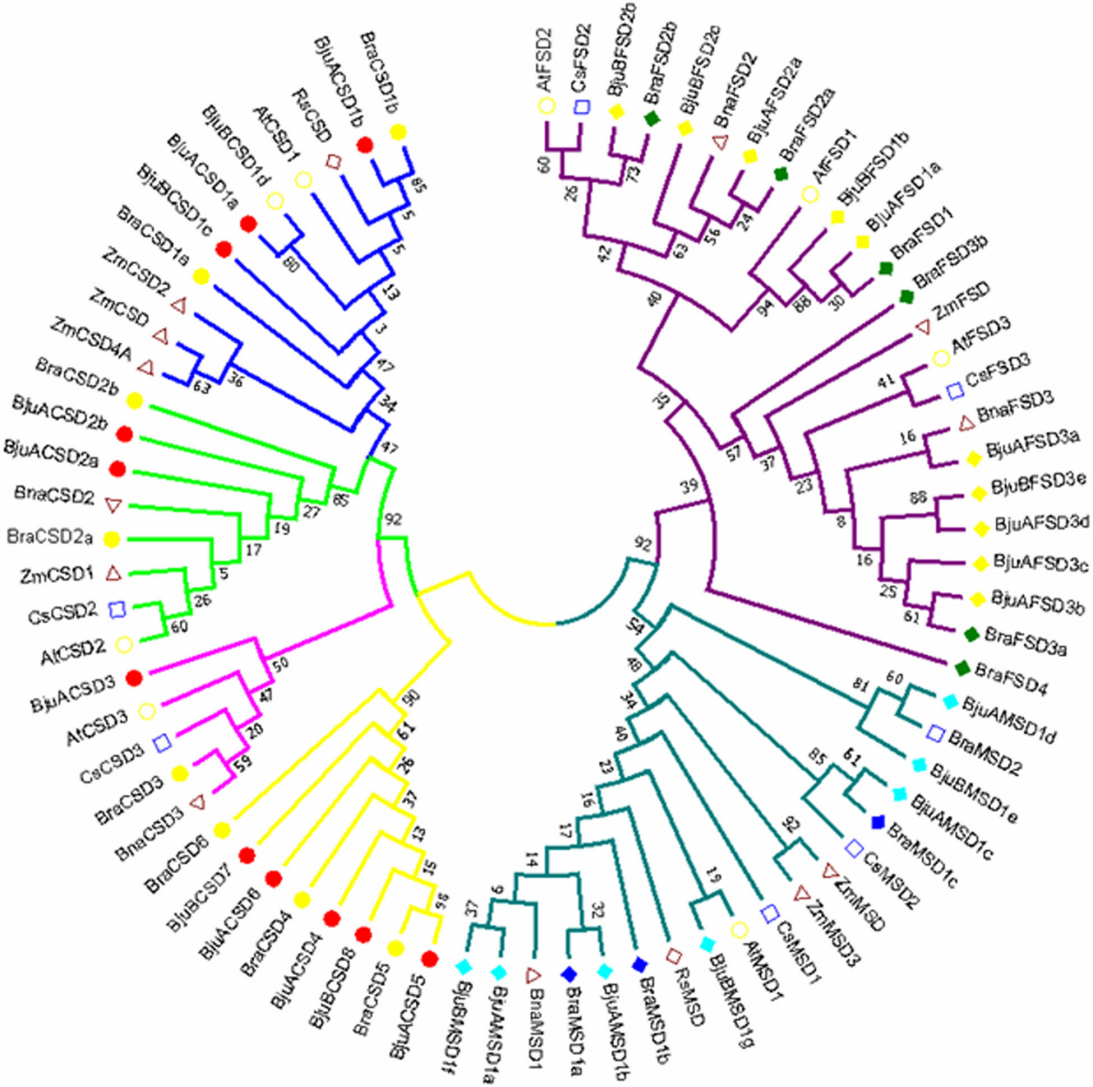
Phyolgenetic tree of *SOD* genes from orthologous species *A. thaliana, B. napus, B. rapa, B. juncea, C. sativa, R. sativus* and *Z. mays*. Neighbor-Joining analysis was performed with a bootstrap value of 1000 using Mega 7 program. Poisson correction method was used to compute the evolutionary distance. Red, yellow, grey, navy blue, sky blue, black and pink colored dots indicate *B. juncea, B. rapa, Z. mays, C. sativa, R. sativus, A. thaliana* and *B. napus SODs* respectively

The additional phylogenetic tree constructed using the reported SOD sequences from *A. thaliana, Z. mays, R. sativus, C. sativus, B. napus* and identified SOD sequences from *B, rapa* and *B. juncea* showed the similar clustering pattern of SOD proteins into two groups CSD and FSD-MSD shown in Fig. 3. *B. juncea* and *B. rapa* showed tight clustering with each other and then with *A. thaliana*. The CSDs and FSD-MSDs were clearly separated into monocot and dicot clades. Also the protein clustering was most likely supported by the sub-cellular predictions made for SODs. For example, *BjuBFSD1b, BjuAFSD1a, BraFSD1* and *AtFSD1* were placed together with a high bootsrap value (95%), all of these proteins are located in the chloroplast.

### Analysis of cis- regulatory elements

**Fig. 4:**
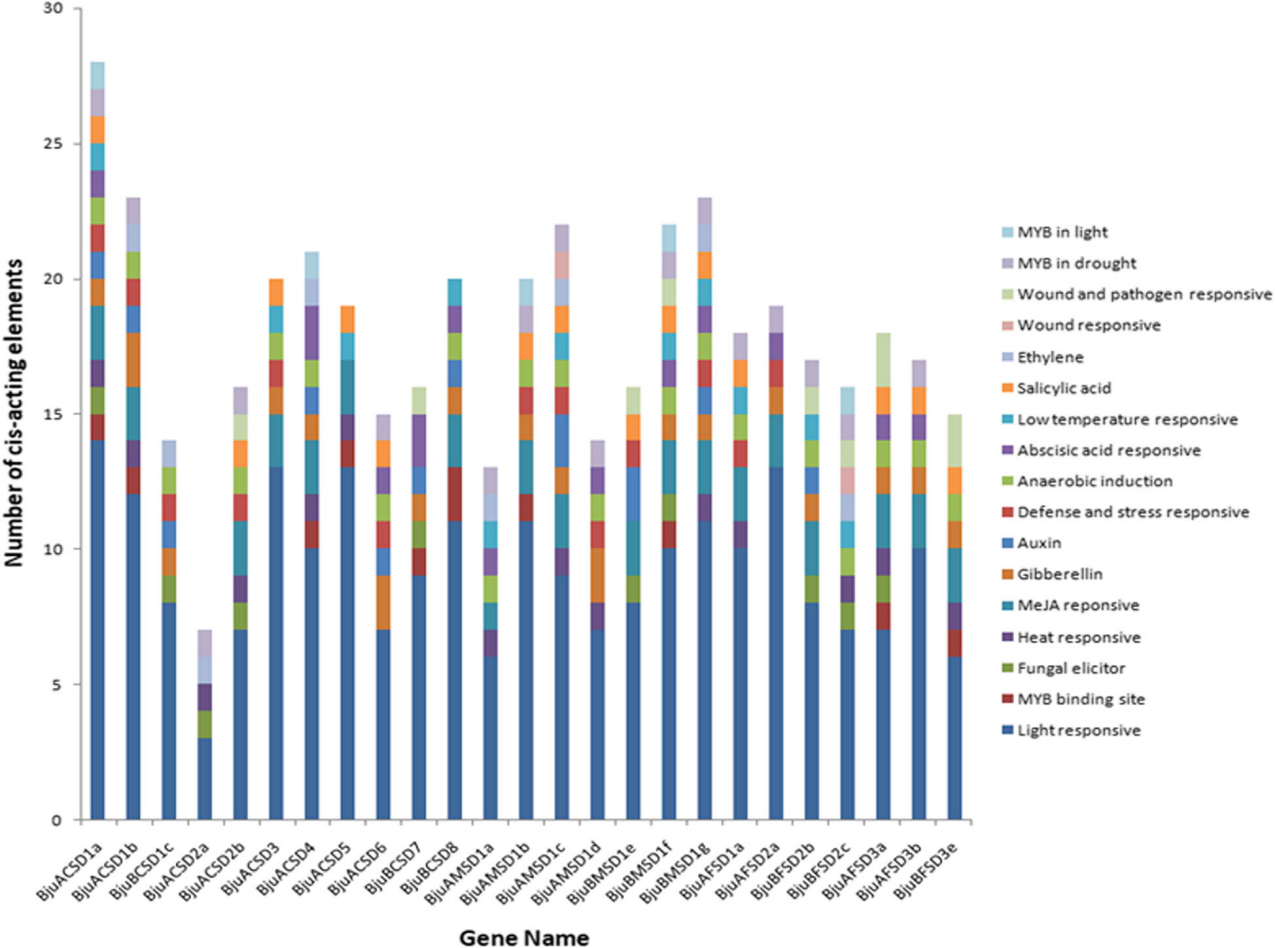
*BjuSOD* promoters showing *cis-*acting regulatory elements responsive to stress. The *cis*-elements with functional similarity are represented by same color coding

Further, the regulatory role of *BjuSODs* was studied by gathering the 1500 bp upstream region of the *SOD* genes from the *B. juncea* from *Brassica* database and the transcriptional response elements of *BjuSOD* were predicted using the PlantCARE tool. All the candidate *BjuSOD* promoters possessed typical CAAT and TATA boxes which are the core cis-acting element in promoter and enhancer regions. The regulatory cis-acting elements likely to be involved in stress responsiveness and transcription factor (TF) binding were also predicted (Fig. 4).

The prediction was grouped into three main types of cis- acting regulatory elements that in one way or the other regulated the gene levels. First, 10 hormone-responsive regulatory elements, ABRE, ERE, TCA-element, TGACG-motif, CGTCA-motif, GARE, P-box, TATC-box, TGA-element, AuxRR-core, which showed relatedness with Abscisic acid (ABA), Ethylene, Salicylic acid (SA), Methyl jasmonate (MeJA), Gibberellin (GA) and Auxin (IAA) responses respectively, were identified in the *BjuSOD* promoters. Second type of regulatory elements found in the *BjSOD* promoters were stress responsive elements. Stress responsive regulatory elements were nine in number namely ARE, MBS, MRE, TC-rich element, HSE, LTR, WUN, Box-W1, W box which were responsive to anaerobic induction, drought inducible, light inducible, defense and stress, heat stress, low temperature stress, wounding, fungal elicitation and wound and pathogen responsive respectively. Third group was the light responsive regulatory elements which comprised of 33 types of different elements predicted in the *BjuSOD* promoters. Various regulatory elements identified in our study prove the fact that the genes also play a crucial role in different types of stress mechanisms.

### Gene expression analysis under abiotic stresses

The expression patterns of abiotic stress responsive *SOD* genes from both *B. juncea* and *B. rapa* species were predicted using the RNA-Seq data available on SRA database of NCBI. The expression analysis was performed under heat, cold, drought and salt stresses. Out of 29 *BjuSOD* and 18 *BraSOD* genes, 10 *BjuSOD* and 14 *BraSOD* genes showed significant differential expression under drought, heat, cold and salt stress, the expression was represented as fold change using heat maps (Additional files 11 and 12). The differentially expressed genes were then validated through qPCR experimentation.

Out of the 10 abiotic stress responsive *BjuSOD* genes, *BjuCSDs* were observed to be up-regulated under all stress conditions, only exception to this was *BjuACSD3* which was found to be down-regulated at 30 DAP with respect to control of cold stress. *BjuFSDs* showed up-regulation under cold and salt stress, whereas the expression under heat and drought was down-regulated with *BjuAFSD3d* being up-regulated in heat stress. *BjuMSD* was down-regulated in both heat and drought stress.

**Fig. 5:**
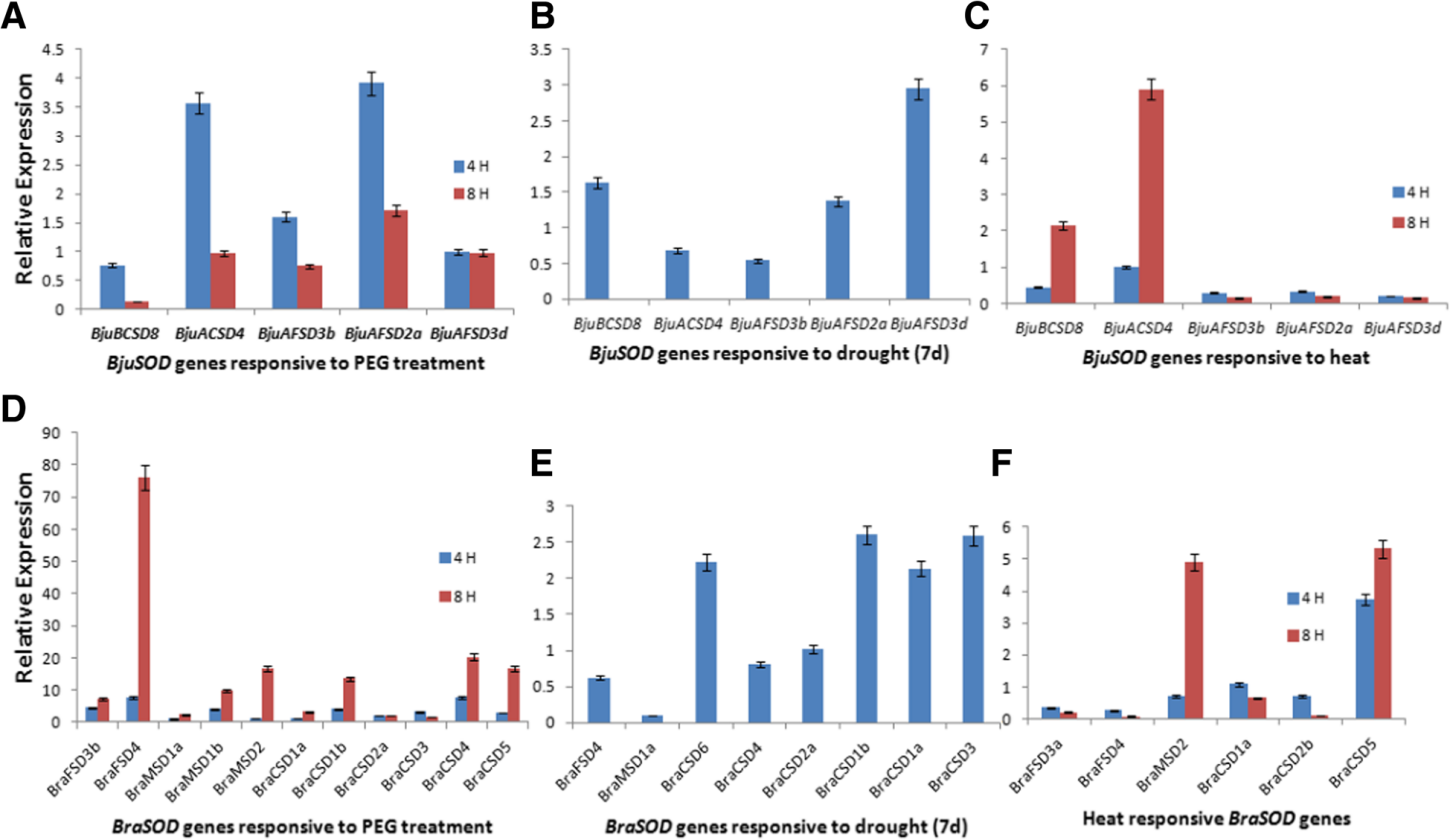
Relative expression level of *BjuSODs* in leaf tissues of *B. juncea* and *B. rapa* under drought stress (**a**, **b**, **d**, **e**) and Heat stress (**c**, **f)** respectively by RT-qPCR. The relative expression was calculated using transcription levels of control leaf tissues as reference

The significant fold change was observed in *BjuACSD4* (3.5 fold), *BjuAFSD3b* (1.6 fold)*, BjuAFSD3d* (1 fold) and *BjuAFSD2a* (3.9 fold) at 4 h drought treatment, whereas at 8 h time point these genes showed decline in the expression levels (Fig. 5a). The fold change observed after 7 day treatment in genes showed continuous decrease in the expression levels of genes *BjuACSD4* (0.6 fold), *BjuAFSD3b* (0.5 fold), *BjuAFSD2a* (1.4 fold). The expression of*BjuAFSD3d* showed an up-regulation of 3 fold after prolonged drought treatment and the expression level of *BjuBCSD8* also showed an up- regulation of 1.6 fold after 7- day treatment (Fig. 5b). The validation of *BjuSOD* genes under heat stress showed the up-regulation of *BjuBCSD8* (2.16 fold) and *BjuACSD4* (5.9 fold) (Fig. 5c), which follows the similar trend as predicted by in silico differential expression analysis. The *BjuFSD* genes showed non-significant fold change but the expression levels were found to show decline when treated for longer duration. The soil moisture content calculated by weight by weight method came out to be 2.15 g.

Total 14 *BraSOD* genes were found to be abiotic stress responsive. The expression profiling of *BraSODs* under drought stress could be grouped into two types based on their expression patterns. First class contained the *BraSODs* which were up-regulated before 8 h of drought stress and after 8 h the expression of these genes were down-regulated upto 12 h of treatment. This group contained *BraFSD4 and BraFSD3a, BraMSD1a and BraMSD1b, BraCSD6 and BraCSD4*. The second group consisted of the genes whose expression was down before 8 h and level increased on continuous drought treatment for up to 12 h. The list included *BraFSD3b, BraCSD1a, 1b, 2a and 3.* Two genes showed decrease in the expression level on increasing time interval of PEG treatment *BraCSD2b* and *BraCSD5.* The gene expression was reversed in case of drought-tolerant variety, wherein all the *BraCSDs* showed reverse expression as compared to the drought-sensitive variety. Only *BraMSD2* similar expression pattern in both tolerant and sensitive variety. The trend observed in expression change under heat stress showed up-regulation of the *BraSODs* after 1 h of heat treatment but then on continuous treatment the expression of all the genes was down-regulated in the heat-tolerant variety, whereas in case of the sensitive variety the expression was up-regulated for upto a period of 6 h of heat treatment but then the expression was down-regulated upto 18 h.

The gene validation through qPCR showed the up-regulation of *BraSOD* genes under drought stress, wherein the increment in expression levels obtained were very high at 8 h heat treatment with fold change of*BraFSD3b* (7.2 fold), *BraFSD4* (76.1 fold), *BraMSD1a* (2.4 fold), *BraMSD1b* (9.9 fold), *BraMSD2* (16 fold), *BraCSD1a* (3.2 fold), *BraCSD1b* (13 fold), *BraCSD2a* (2.12 fold), *BraCSD4* (20 fold) and *BraCSD5* (16 fold). The down-regulation was observed in the expression level of *BraCSD3* from 3.2 fold to 1.6 fold when treated for 8 h (Fig. 5d). The genes showed decline in the expression level when treated for 7 days of drought with significant fold changes observed in *BraCSD6* (2.2 fold), *BraCSD2a* (1 fold), *BraCSD1b* (2.6 fold), *BraCSD1a* (2.1 fold), *BraCSD3* (2.6 fold) (Fig. 5e). The expression levels obtained for heat stress showed an up-regulation in *BraMSD2* (4.9 fold) and *BraCSD5* (5.3 fold). *BraCSD1a* showed a decline from 1 fold to approximately half on prolonged heat treatment (Fig. 5f).

## Discussion

*B. juncea* and *B. rapa* being the major oilseed and vegetable crops of the world, are affected by various abiotic and biotic stresses leading to the downfall in the crop yield [48, 49]. Reactive oxygen species (ROS) produced under normal and stress conditions are controlled and scavenged by SODs, which constitute the first line of defence against ROS [50]. SODs are involved in various abiotic and biotic stress related responses [18, 19, 51]. Complete characterization of *SOD* gene family in *B. juncea* and *B. rapa* has not been reported. The availability of whole genome sequence of both the species aided in genome-wide characterization of the *SOD* genes, which may further be used to improve the crop yield on field.

The basic pipeline for identifying *SOD* genes including Blast search the known proteins of related families and pfam search, was followed as reported earlier for other plants [18, 19, 44]. A total of 29 *SODs* (12 CSDDs, 7 MSDs and 10 FSDs) in *B. juncea* and 18 *SODs* (8 CSDs, 4 MSDs and 6 FSDs) in *B. rapa* were identified following the above mentioned procedure. Extended *SOD* gene number in *B. juncea* could be owed to the tetraploid nature (AABB, 2n = 36) of the genome. Previously 7 *SODs* have been reported in *A. thaliana* [17]*,* 8 in *Sorghum bicolor* [44], 18 in *G. hirsutum* [16] and 23 in *T. aestivum* [18]. Also the high gene number in *B. juncea* was similar to the findings in polyploid plants like *G. hirsutum.* The gene number identified in *B. rapa* also follow the same trend as in the diploid plants like Arabidopsis*.*

*SOD* genes in *B. juncea* have been derived from both subgenomes (A and B). The identified genes were clustered into two main groups: CSD and MSD-FSD. Out of the 29 genes for *B. juncea* 16 genes (8 CSD, 4 MSD and 4 FSD) were from A sub-genome and 9 genes (3 CSD, 3 MSD and 3 FSD) were from B sub-genome. The 18 *SOD* genes of *B. rapa* were widely distributed across the A genome. A9 chromosome had the highest number of genes (4 genes), followed by chromosomes A4, A6, A1 and A3 (each with 2 genes), remaining genes were located on A5, A8 and A10 each with single gene. Whole genome duplication (WGD) and polyploidy events might have contributed to the *SOD* number in the *Brassica* species*.*

Gene architecture examination revealed that the *SOD* genes comprised of a variable intron number, wherein the range varied from 1 to 9 in *BjuCSDs* and *BjuFSDs*, and BjuMSDs whereas the intron number ranged from 2 to 7 in *BraSODs*. Previous reports suggest the conserved intron pattern for cytosolic and chloroplastic CSD, that cytosolic genes contain seven introns and chloroplastic contain eight introns [52]. We observed that only three *SODs* (*BjuSOD4*, *BjuBFSD2c* and *BjuAFSD2a*) included seven introns, whereas the intron number varied in rest of the genes as reported in *Sorghum bicolor* and *S. lycopersicum* [19, 44]. The exon/intron gain/loss, insertion/deletion and exonization/pseudoexonization leads to the variation in exon/intron number and also results in structural variability in various genes [44].

Maximum number of introns were found to be in 0 phase followed by 1 phase. The distribution of intron phase is conserved during the evolutionary process. The 0 phase introns occur most frequently while the 2 phase introns occur least frequently. The introns occurring in 1 phase suggest that the 1st and 2nd base in the codon is interrupted by the intron, whereas phase 2 intron suggest the appearance of intron between the2^nd^ and 3rd base of the codon [53].

The physico-chemical properties like amino acid length, molecular weight and pI of the SOD proteins analyzed in *B. rapa* and *B juncea* showed a similar trend as those described earlier in plants like *S. bicolor* [44]. Plants usually contain CSDs in their cytoplasm, chloroplast and peroxisome [44, 54]. FSDs have been reported to be found in chloroplast and cytoplasm in cowpea [55, 56]. MSDs were associated with the mitochondrion. The sub-cellular location predicted for BjuSOD and BraSOD proteins was similar to the previously reported studies.

SOD proteins are highly conserved in nature [57], thus conserved motifs were identified for both CSD and MSD-FSD proteins in both *B. juncea* and *B. rapa* species. SOD proteins contain Cu-ZnSOD domain (pfam00080) in CSDs, iron/manganese alpha-hairpin domain (pfam00081) and iron/manganese C-terminal domain (pfam02777) in FSD-MSDs. Further additional domains like HMA domain, Helix-loop-helix domain, Ribosomal_S26e domain were also found to be associated with the SOD proteins. Presence of HMA domain has previously been reported in *S. lycopersicum* and *T. aestivum* [18, 19].

The structural framework is important for the perfect functioning of proteins and thus the primary, secondary and tertiary structures of BjuSOD and BraSOD proteins were examined using the earlier reported SOD proteins in the PDB database [45–47]. The activity of CSD or FSD-MSDs depends on the presence of the metal co-factor in its vicinity (Cu-Zn or Fe-Mn). The metal deficiency results in the inhibition of the isozyme dependant on that metal co-factor, but elevation of the other type of isozyme. Study conducted on *Pisum sativum* indicated an increase in the Cu-ZnSOD activity in the absence of Mn due to the subsequent decrease in the level of MnSOD isozyme [58]. Another study reported an increase in the FeSOD and MnSOD activity in the Cu deficiency in pea plant suggesting that the isozyme biosynthesis is an interdependent and a co-ordinated process, wherein the concentration of the metal cofactors decide the balance between the isozymes [59]. SODs show high conservation pattern across vast number of organisms, similarly primary structures of Cu-ZnSODs and Fe-MnSODs were aligned which exhibited the conservation pattern with respect to the active site residues and metal binding sites as observed in other plants marking the functional importance of the SOD proteins [18].

The basic CSD structure is a β-barrel composed of eight anti-parallel β-strands organized in a Greek key pattern, stabilizing the overall protein structure [45]. Leu residue fills the ends of β-barrels and a species specific variable loop between β2-β3 can be seen in the CSD structure [57]. A single disulphide bridge is found between two conserved cys residues which stabilize the structure [45]. The Cu^2+^ ion is liganded by four His residues out of which one is present out of the β-barrel structure, with rest in the barrel. Zn^2+^ ion is liganded by three His residues and one Asp, one of the His is common between both Cu^2+^ and Zn^2+^ ion. The active metal site lies on the external side of the β-barrel [45, 57].

The secondary and tertiary MSD and FSD structures were also in congruence with the known protein structures. The FSD and MSDs are considered to act in a cambialistic was where either Fe or Mn can be taken up by the SOD for its activity [60]. The structure is composed of α-helices and anti-parallel β-sheets. Differences have been observed at minor levels between archae FSD and Human MSD wherein there is a substitution of the tryptophan by an alanine of the shell forming residues due to which the distance between the hydroxide ion and side-change is shortened [47]. The active site is liganded by two His residues from N-terminus and two residues one His and another Asp from the C-terminus, and an axial coordinated solvent which Hydrogen bonds with the conserved Gln (Gln 69 in FeSOD and Gln146 in MnSOD) [61]. Both protein groups exhibit very high sequence similarity.

Phylogenetic analysis revealed a close relatedness between CSD and MSD-FSD members, however both formed two separate groups based on the bootstrap values. The chloroplastic FeSODs and mitochondrial MnSODs were clustered into two different groups, based on the earlier reports that the two SODs shared a common ancestor but then a significant divergence was observed in the amino acid composition of both the groups [56]. The phylogenetic tree created using *BjuSODs* and *BraSODs* clearly depicted the evolutionary relatedness between *juncea* and *rapa.* Many genes showed greater than 95% identity like *BraCSD5* and *BjuACSD5*. Also the *BjuSODs* and *BraSODs* that were clustered together in the same phylogenetic tree with a high bootstrap value showed similar intron number which explains that these genes have a conserved pattern. The *SODs* which were clustered together in the *rapa-juncea* phylogram also shared similar chromosomal occurrence on the A genome, which also explains the fact that during evolution these genes might have been derived by *B. juncea* from the progenitor species the progenitor species *B. rapa.* Furthermore, related *SOD* orthologous genes from various plants were clustered together, but the monocot and dicot clades showed clear separation. The distinct evolutionary relationship of *SODs* in monocots and dicots can be explained on the basis of common ancestory of both groups of plants [62].

Abiotic factors like drought, heat, cold, salinity pose a serious threat to the crop yield of *Brassica* crops, thus expression analysis of *SOD* genes under drought and heat stress were studied. Previous reports on *G. hirusutm, A. thaliana, S. lycopersicum* suggest the roles that *SODs* play in overcoming the stress [16, 17, 19]. Transgenic cassava plants expressing high levels of Cu-ZnSOD and Ascorbate peroxidase provided tolerance to chilling stress [3]. Studies were also conducted to test the changes in the levels of different isozymes of SODs under various abiotic stresses. Salt stress increased the activity of Cu-ZnSOD in the leaves of *Citrus limonum*, low temperature induced the increased SOD and catalase activity in *Avenanuda* plant [62, 63]. These studies suggest that *SODs* play an eminent role in overcoming the abiotic stress. In our study the expression levels were studied under drought and heat stress in both *B. rapa* and *B. juncea* to understand their involvement in stress responsiveness. The *BjuSOD* genes tested for drought stress showed similar trend as predicted by in-silico analysis. When the plants were exposed to longer duration of drought stress, two genes *BjuBCSD8* and *BjuAFSD3d* showed up-regulation in the expression levels. The other three genes showed continuous decrease in the expression level from 4 h to 7 day drought treatment. On exposure to heat, *BjuACSD4* and *BjuBCSD8* showed significant increase in the expression levels that follows the in-silico prediction, whereas the other three genes showed decrease in the expression but that change was not significant. *B. rapa* plants when exposed to drought showed increment in the expression level in the initial period i.e. from 4 h to 8 h in all genes except for *BraCSD3* that showed decline in the level and this data also follows the similar trend described by in-silico analysis. But when exposed to drought for 7 day period the expression level was found to be decreased in all the *SODs.* Heat treated *rapa* samples showed significant changes in *BraMSD2* and *BraCSD5.*

*Cis*-acting regulatory elements serve as important molecular switches involved in transcriptional regulation of the gene activities controlling various biological processes like abiotic stress responses, hormone responses and developmental processes [64]. In *B. juncea* several stress responsive regulatory elements were identified namely ARE, MBS, MRE, TC-rich element, HSE, LTR, WUN, Box-W1, W box. The transcription factors interact with the cis regulatory elements and activate the stress tolerance mechanism like ERF transcription factor binds the GCC-box and provide tolerance to salt stress in tomato [65]. The identified regulatory elements in our study help in understanding their role in the various abiotic and biotic stress related mechanisms.

## Conclusion

In our study, the reported *B. juncea* and *B. rapa* genomes were analyzed for the identification of *SOD* genes. The *SODs* were classified into three types of plant *SODs* (*Cu-ZnSOD, FeSOD* and *MnSOD*) which were widely distributed in the genome. The extended *SOD* family could be due to the WGD and polyploidisation events experienced by the *Brassica* genome. The *SOD* specific distinctive features like exon-intron organization, motifs, sub-cellular localization and functional analysis were also explored in the study. Stress responsive and hormonal responsive *cis-*regulatory elements in the promoter regions of *SOD* genes were also identified which varied in types and number in *SOD* genes. Further, differentially expressed genes responsive to abiotic stresses in *B. rapa* and *B. juncea* were detected using the RNA-seq and qPCR data, and we suggested that different *SODs* show different expression patterns under different abiotic conditions at different time points. Thus these genes can be targeted for crop improvement. The expression study also explained the role of *SODs* in overcoming abiotic stress in *Brassica* species.

## Additional files

Additional file 1: Primer list used for RT-qPCR validation of abiotic-stress responsive *SOD* genes in *B. juncea* and *B. rapa. (DOCX 16 kb)*

Additional file 2: **a** Figure depicts the multiple sequence alignment of Cu-ZnSODs from *B. juncea*, anti-parallel β-strands are shown by over head red arrows, Cu binding residues are shown in yellow asterisk, Zn residues in blue asterisk, Bridging histidine is represented by two asterisk. Also various important structural elements are shown like V-loop (Variable loop with over head yellow line), Electrostatic loop (Green) and Greek key loops 1 and 2 (GK1 and GK2 in purple). The Cu^2+^ and Zn^2+^ motif signature sequence is highlighted in black box. **b** Figure shows the multiple sequence alignment of MnSODs from *B. juncea,* wherein their metal binding sites is highlighted in black box and the metal binding residues are marked with purple asterisks. Also the α-helices and β-sheets are shown by over head black arrows. **c** Figure shows the multiple sequence alignment of FeSODs from *B. juncea,* wherein their metal binding sites is highlighted in black box and the metal binding residues are marked with yellow asterisks. Also the α-helices and β-sheets are shown by over head black arrows. (ZIP 2875 kb)

Additional file 3: **a** Figure depicts the multiple sequence alignment of Cu-ZnSODs from *B. rapa*, anti-parallel β-strands are shown by over head red arrows, Cu binding residues are shown in yellow asterisk, Zn residues in blue asterisk, Bridging histidine is represented by two asterisk. Also various important structural elements are shown like V-loop (Variable loop with over head yellow line), Electrostatic loop (Green) and Greek key loops 1 and 2 (GK1 and GK2 in purple). The Cu^2+^ and Zn^2+^ motif signature sequence is highlighted in black box. **b** Figure shows the multiple sequence alignment of MnSODs from *B. rapa,* wherein their metal binding sites is highlighted in black box and the metal binding residues are marked with blue asterisks. Also the α-helices and β-sheets are shown by over head black arrows. **c** Figure shows the multiple sequence alignment of FeSODs from *B. rapa,* wherein their metal binding sites is highlighted in black box and the metal binding residues are marked with pink asterisks. Also the α-helices and β-sheets are shown by over head black arrows. (ZIP 2377 kb)

Additional file 4: Three dimensional structure of Cu-ZnSOD determined using PyMOL shows the occurrence of eight anti-parallel β-sheets and two α-helices (A). Cu^2+^ and Zn^2+^ binding residues are shown in blue and pink colored sticks with the bridging histidine highlighted with red stick and the disulphide bond formed by two Cys-Cys is shown in cyan color (B). (TIF 347 kb)

Additional file 5: Three dimensional structure of MnSOD determined using PyMOL shows the occurrence of three anti-parallel β-sheets and seven α-helices (A). The metal binding residues are shown in blue sticks which form a bipyramidal structure in the presence of H_2_O. Also the conserved Glu residue is marked with pink sphere (B). (TIF 539 kb)

Additional file 6: Three dimensional structure of FeSOD determined using PyMOL shows the occurrence of three anti-parallel β-sheets and nine α-helices (A). The metal binding residues are shown in blue sticks which form a bipyramidal structure in the presence of H_2_O (B). Also the shell forming residues are highlighted with pink sticks (C). (TIF 457 kb)

Additional file 7: Motif sequences of (a) BjuSOD and (b) BraSOD proteins identitifed by MEME tool. (DOCX 14 kb)

Additional file 8: Conserved motif logos in (a) BjuSOD and (b) BraSOD proteins. (TIF 530 kb)

Additional file 9: Functional classification of (a) *BjuSOD* and (b) *BraSOD* genes on the basis of Gene Ontology (GO) terms assigned to various genes using BLAST2GO tool. GO terms enrichments in 3 different categories i.e. i) Cellular Component, ii) Molecular Function and iii) Biological Process were predicted. (TIF 126 kb)

Additional file 10: Phyolgenetic tree of *SOD* genes from *B. rapa* and *B. juncea.* Neighbor-Joining analysis was performed with a bootstrap value of 1000 using Mega 7 program. Poisson correction method was used to compute the evolutionary distance. (TIF 141 kb)

Additional file 11: Expression analysis of *BjuSOD* genes under abiotic stresses. The differential expression profile is shown by Heat map under cold stress (A), Heat and drought stress (B), Salt stress (C). The HCE3.5 software was used to cluster together the genes showing similar expression pattern. (TIF 139 kb)

Additional file 12: Expression analysis of *BraSOD* genes under abiotic stresses. The differential expression profile is shown by Heat m ap under drought stress (DS) in drought sensitive variety (A) and drought tolerant variety (B); Heat stress in Heat sensitive variety (C) and Heat tolerant variety (D). The HCE3.5 software was used to cluster together the genes showing similar expression pattern. (TIF 190 kb)

## Abbreviations

°C: Degree Celsius
aa: Amino acid
ABA: Abscisic acid
ACT7: Actin-7
BRAD: *Brasssica* database
CAT: Catalase
CDD: Conserved Domain Database
cDNA: Complementary deoxyribonucleic acid
CSD: Copper-zinc superoxide dismutase
CZ: Cu/Zn-superoxide dismutase domain
DAP: Days After Pollination
FAO: Food and Agriculture Organization
FPKM: Fragments per kilobase per million reads
FSD: Iron superoxide dismutase
GA: Gibberellin
Gar1: Gar1/Maf1 RNA binding region
gm: gram
GO: Gene Ontology
GSDS: Gene Structure Display Server
GSHPx: Glutathione peroxidase domain
h: hour
HLH: Helix-loop helix domain
HMA: Heavy metal associated domain
HMM: Hidden Markov Model
IAA: Auxin
IMA: Fe/Mn-SODs, alpha-hairpin domain
IMC: Fe/Mn-SODs, C-terminal domain
I-TASSER: Iterative Threading ASSEmbly Refinement
kDa: kilo Dalton
MAFFT: Multiple Alignment using Fast Fourier Transform
MeJA: Methyl jasmonate
MEME: Multiple Expectation Maximization for Motif Elicitation
MSD: Manganese superoxide dismutase
MUSCLE: Multiple Sequence Comparison by Log-Expectation
mw: Molecular weight
N_2_: Nitrogen
NCBI: National Centre for Biotechnology Information
P_C14: Peptidase_C14 domain
PEG: Polyethylene glycol
p*I*: Isoelectric point
POX: Peroxidase
qRT-PCR: Quantitative Real Time Reverse Transcription Polymerase Chain Reaction
R_L18p: Ribosomal_L18p
R_S26e: Ribosomal_S26e
RNA: Ribonucleic acid
ROS: Reactive oxygen species
RSEM: RNA-Seq by Expectation Maximization
RVT-3: Reverse Transcriptase 3
SA: Salicylic acid
SMART: Simple Modular Architecture Research Tool
SOD: Superoxide dismutase
SRA: Sequence Read Archive
TF: Transcription factor
TIPS-41: Tonoplastic Intrinsic Protein-41
*w*/*v*: weight/volume
WGT: Whole genome triplication
