# Supplementary material for: Genome-wide identification and characterization of abiotic-stress responsive SOD (superoxide dismutase) gene family in Brassica juncea and B. rapa
Source: BMC Genomics. 2019 Mar 19;20:227. doi: 10.1186/s12864-019-5593-5 (PMC6425617; doi:10.1186/s12864-019-5593-5)
Supplement: Supplementary file 1 — Primer list used for RT-qPCR validation of abiotic-stress responsive SOD genes in B. juncea and B. rapa. (DOCX 16 kb) [file 12864_2019_5593_MOESM1_ESM.docx]

**Additional file 1. Primer list used for RT-qPCR validation of abiotic-stress responsive *SOD* genes in *B. juncea* and *B. rapa.***

| Gene ID | Primer Sequence | Tm |
| --- | --- | --- |
| BjuAFSD3b F | GAAGAGGGAAGAGAGGAGACTTG | 57° C |
| BjuAFSD3b R | TGTACTTAGCCCTCTCGTTCTTG | 57° C |
| BjuAFSD2a F | TAGCTGTTTCTGGTGTTGTCAGA | 55° C |
| BjuAFSD2a R | GTAGCACAACCTCTTCCAATGAC | 55° C |
| BjuAFSD3d F | GGAAGAGCTTATTAAGGCCACAT | 55° C |
| BjuAFSD3d R | CCAAACTGAGTAAGAGCTGCATT | 55° C |
| BjuACSD4 F | CACTTCAAGATCATACCCACACA | 56° C |
| BjuACSD4 R | CAATTAGATCCACAACCTTGAGC | 56° C |
| BjuBCSD8 F | CAGACATCTTTGGAGTGGTAAGG | 53° C |
| BjuBCSD8 R | AGCAACCTTGAGCTTCTCTTTCT | 53° C |
| BraMSD1a F | CTCTCTTGAAGGTTTGGTGAAG | 55° C |
| BraMSD1a R | CAAACATCTATACCCACCAGAGG | 55° C |
| BraFSD4 F | GTGTCATGGGAAACTGTAAGCAC | 57° C |
| BraFSD4 R | TAAACCTCTGGCTCTTCATCATC | 57° C |
| BraFSD3a F | GAAGAGGGAAGAGAGGAGACTTG | 59° C |
| BraFSD3a R | TGTACTTAGCCCTCTCGTTCTTG | 59° C |
| BraCSD6 F | GGGGTAGTAAGATTTGCTCAGGT | 58° C |
| BraCSD6 R | GGCTCTGTGATTGTATGGTCTTC | 58° C |
| BraCSD4 F | CACTTCAAGATCATACCCACACA | 57° C |
| BraCSD4 R | CAATTAGATCCACAACCTTGAGC | 57° C |
| BraCSD2a F | TCAACCCTAACAACATGACACAC | 56° C |
| BraCSD2a R | CAGAGTTAGGACCAGTCAGAGGA | 56° C |
| BraCSD1b F | ACTATCTTCTTCACCCAGGAAGG | 57° C |
| BraCSD1b R | GCCCTTCCTACAATAGAGTTTGG | 57° C |
| BraCSD1a F | ATTCACACTCGACGTCTTCATCT | 55° C |
| BraCSD1a R | AGCTCTGATCTTCCCTTCAAACT | 55° C |
| BraCSD3 F | CTGCAACTCTACTGGACCTCACT | 55° C |
| BraCSD3 R | GTCTGATCCAGCGAAAATGTTAC | 55° C |
| BraCSD2b F | TTGGTGTGTAATAAAGGAGAAGGTG | 52° C |
| BraCSD2b R | AAGTGGATTGGGTAAATGAGAGATG | 52° C |
| BraCSD5 F | CAGTTGGTGTATCAATGAGTATGGA | 57° C |
| BraCSD5 R | CCTTCATCTTCTCTTTCTTTACCGT | 57° C |
| BraMSD2 F | ATTACCTCTAGCAAGATTCCTTCCT | 52° C |
| BraMSD2 R | CTTCTTAGGCTATTTGTCACCCATC | 52° C |
| BraMSD1b F | TGAAGGTTTGGTGAAGAAGATGAG | 57° C |
| BraMSD1b R | ACTTTCCACACGTTCTTCAGATAC | 57° C |
| BraFSD3b F | AAGAGGGAAGAGAGAAGACTTGAA | 57° C |
| BraFSD3b R | AACGTGTTTATGTACTTTCCCCTC | 57° C |
| TTIPS-41 F | TGAAGAGCAGATTGATTTGGCT | 54° C |
| TIPS-41 R | ACACTCCATTGTCAGCCAGTT | 54° C |
| ACT-7 F | CTACGAGTTACCTGATGGA | 54° C |
| ACT-7 R | ATGATGGAGTTGTAAGTTGTC | 54° C |
