# Supplementary figures and images for: Genome-wide identification and characterization of abiotic-stress responsive SOD (superoxide dismutase) gene family in Brassica juncea and B. rapa
Source: BMC Genomics. 2019 Mar 19;20:227. doi: 10.1186/s12864-019-5593-5 (PMC6425617; doi:10.1186/s12864-019-5593-5)

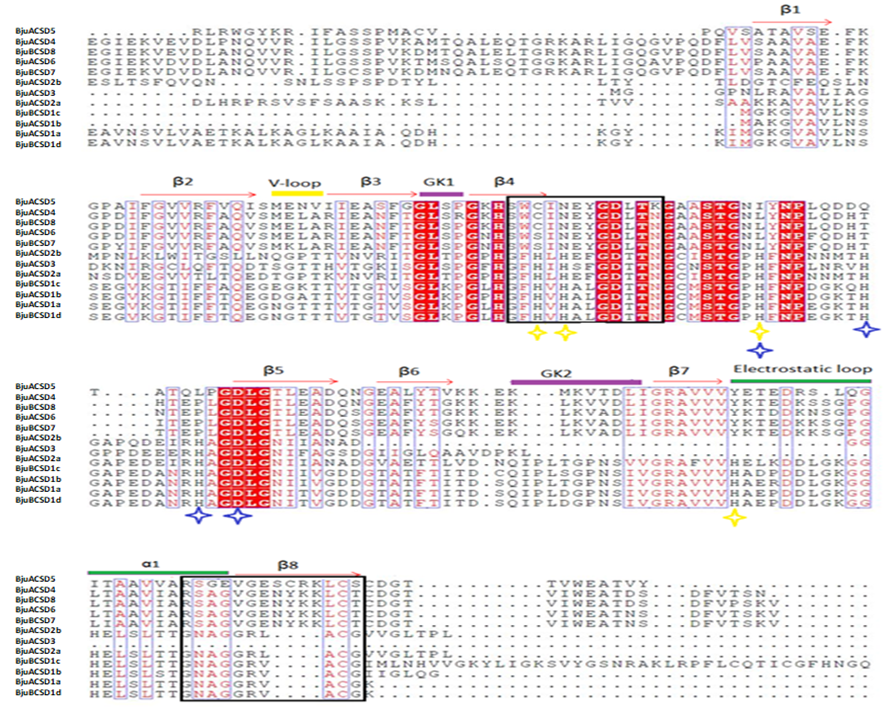

Supplement: Supplementary file 2 — a Figure depicts the multiple sequence alignment of Cu-ZnSODs from B. juncea, anti-parallel β-strands are shown by over head red arrows, Cu binding residues are shown in yellow asterisk, Zn residues in blue asterisk, Bridging histidine is represented by two asterisk. Also various important structural elements are shown like V-loop (Variable loop with over head yellow line), Electrostatic loop (Green) and Greek key loops 1 and 2 (GK1 and GK2 in purple). The Cu2+ and Zn2+ motif signature sequence is highlighted in black box. b Figure shows the multiple sequence alignment of MnSODs from B. juncea, wherein their metal binding sites is highlighted in black box and the metal binding residues are marked with purple asterisks. Also the α-helices and β-sheets are shown by over head black arrows. c Figure shows the multiple sequence alignment of FeSODs from B. juncea, wherein their metal binding sites is highlighted in black box and the metal binding residues are marked with yellow asterisks. Also the α-helices and β-sheets are shown by over head black arrows. (ZIP 2875 kb) [file 12864_2019_5593_MOESM2_ESM.zip › Additional file 6a.tif]

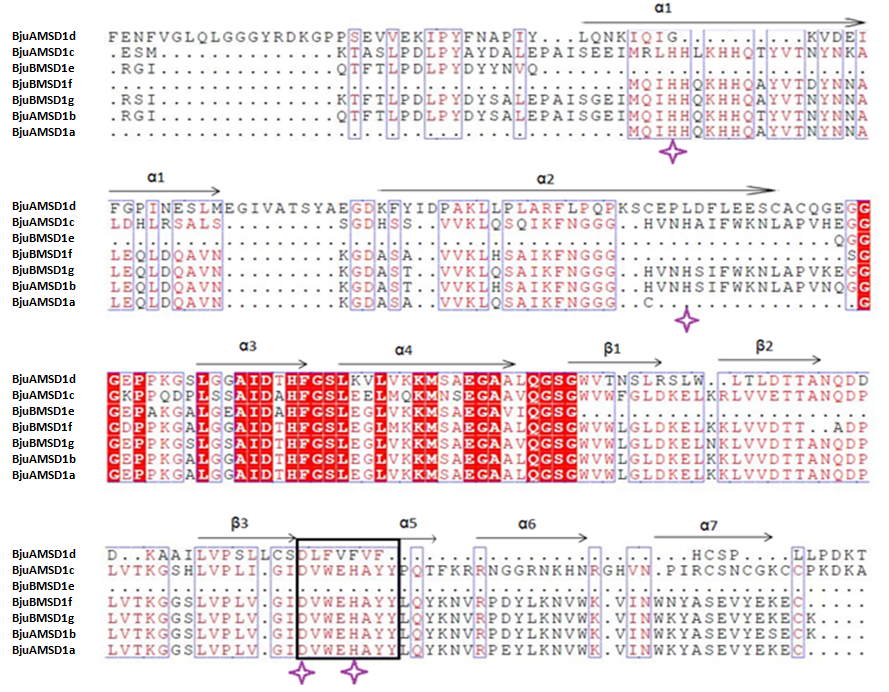

Supplement: Supplementary file 2 — a Figure depicts the multiple sequence alignment of Cu-ZnSODs from B. juncea, anti-parallel β-strands are shown by over head red arrows, Cu binding residues are shown in yellow asterisk, Zn residues in blue asterisk, Bridging histidine is represented by two asterisk. Also various important structural elements are shown like V-loop (Variable loop with over head yellow line), Electrostatic loop (Green) and Greek key loops 1 and 2 (GK1 and GK2 in purple). The Cu2+ and Zn2+ motif signature sequence is highlighted in black box. b Figure shows the multiple sequence alignment of MnSODs from B. juncea, wherein their metal binding sites is highlighted in black box and the metal binding residues are marked with purple asterisks. Also the α-helices and β-sheets are shown by over head black arrows. c Figure shows the multiple sequence alignment of FeSODs from B. juncea, wherein their metal binding sites is highlighted in black box and the metal binding residues are marked with yellow asterisks. Also the α-helices and β-sheets are shown by over head black arrows. (ZIP 2875 kb) [file 12864_2019_5593_MOESM2_ESM.zip › Additional file 6b.tif]

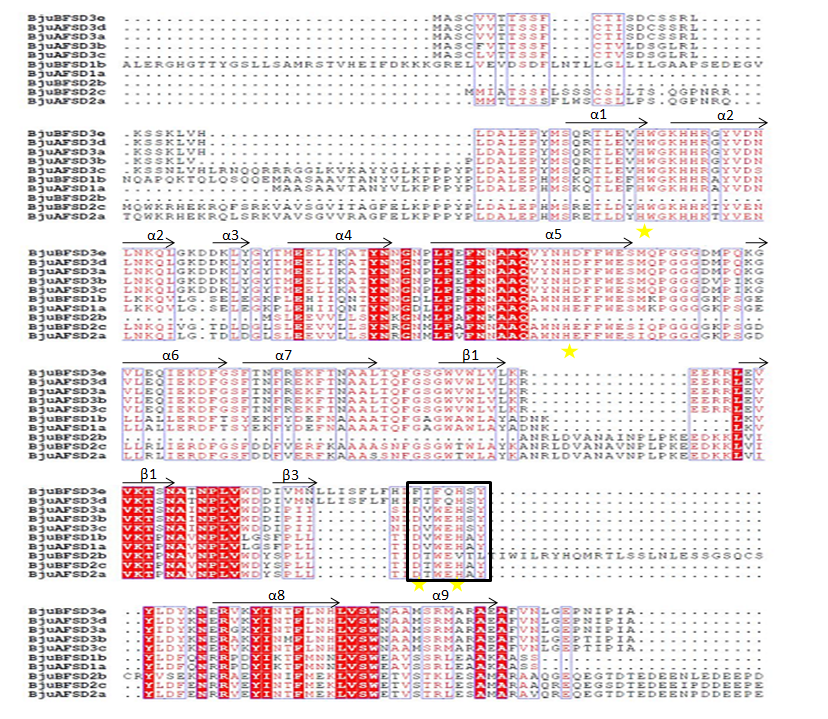

Supplement: Supplementary file 2 — a Figure depicts the multiple sequence alignment of Cu-ZnSODs from B. juncea, anti-parallel β-strands are shown by over head red arrows, Cu binding residues are shown in yellow asterisk, Zn residues in blue asterisk, Bridging histidine is represented by two asterisk. Also various important structural elements are shown like V-loop (Variable loop with over head yellow line), Electrostatic loop (Green) and Greek key loops 1 and 2 (GK1 and GK2 in purple). The Cu2+ and Zn2+ motif signature sequence is highlighted in black box. b Figure shows the multiple sequence alignment of MnSODs from B. juncea, wherein their metal binding sites is highlighted in black box and the metal binding residues are marked with purple asterisks. Also the α-helices and β-sheets are shown by over head black arrows. c Figure shows the multiple sequence alignment of FeSODs from B. juncea, wherein their metal binding sites is highlighted in black box and the metal binding residues are marked with yellow asterisks. Also the α-helices and β-sheets are shown by over head black arrows. (ZIP 2875 kb) [file 12864_2019_5593_MOESM2_ESM.zip › Additional file 6c.tif]

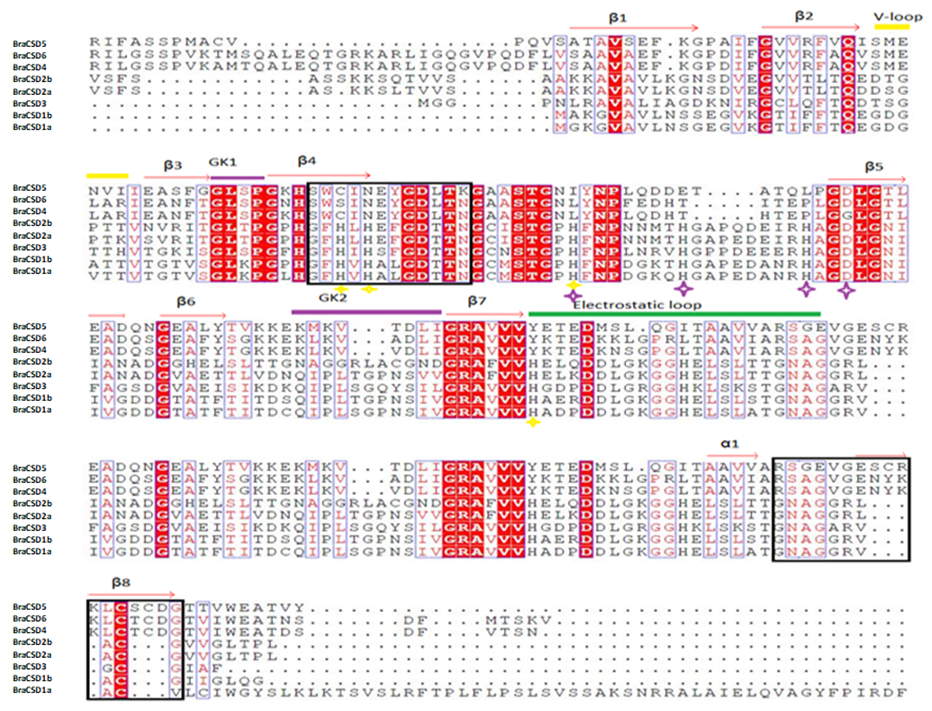

Supplement: Supplementary file 3 — a Figure depicts the multiple sequence alignment of Cu-ZnSODs from B. rapa, anti-parallel β-strands are shown by over head red arrows, Cu binding residues are shown in yellow asterisk, Zn residues in blue asterisk, Bridging histidine is represented by two asterisk. Also various important structural elements are shown like V-loop (Variable loop with over head yellow line), Electrostatic loop (Green) and Greek key loops 1 and 2 (GK1 and GK2 in purple). The Cu2+ and Zn2+ motif signature sequence is highlighted in black box. b Figure shows the multiple sequence alignment of MnSODs from B. rapa, wherein their metal binding sites is highlighted in black box and the metal binding residues are marked with blue asterisks. Also the α-helices and β-sheets are shown by over head black arrows. c Figure shows the multiple sequence alignment of FeSODs from B. rapa, wherein their metal binding sites is highlighted in black box and the metal binding residues are marked with pink asterisks. Also the α-helices and β-sheets are shown by over head black arrows. (ZIP 2377 kb) [file 12864_2019_5593_MOESM3_ESM.zip › Additional file 7a.tif]

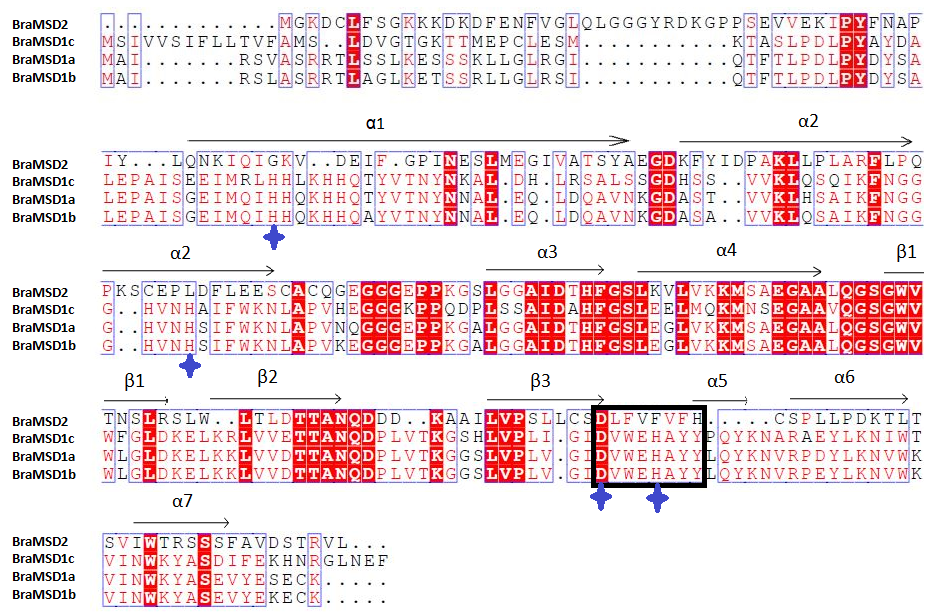

Supplement: Supplementary file 3 — a Figure depicts the multiple sequence alignment of Cu-ZnSODs from B. rapa, anti-parallel β-strands are shown by over head red arrows, Cu binding residues are shown in yellow asterisk, Zn residues in blue asterisk, Bridging histidine is represented by two asterisk. Also various important structural elements are shown like V-loop (Variable loop with over head yellow line), Electrostatic loop (Green) and Greek key loops 1 and 2 (GK1 and GK2 in purple). The Cu2+ and Zn2+ motif signature sequence is highlighted in black box. b Figure shows the multiple sequence alignment of MnSODs from B. rapa, wherein their metal binding sites is highlighted in black box and the metal binding residues are marked with blue asterisks. Also the α-helices and β-sheets are shown by over head black arrows. c Figure shows the multiple sequence alignment of FeSODs from B. rapa, wherein their metal binding sites is highlighted in black box and the metal binding residues are marked with pink asterisks. Also the α-helices and β-sheets are shown by over head black arrows. (ZIP 2377 kb) [file 12864_2019_5593_MOESM3_ESM.zip › Additional file 7b.tif]

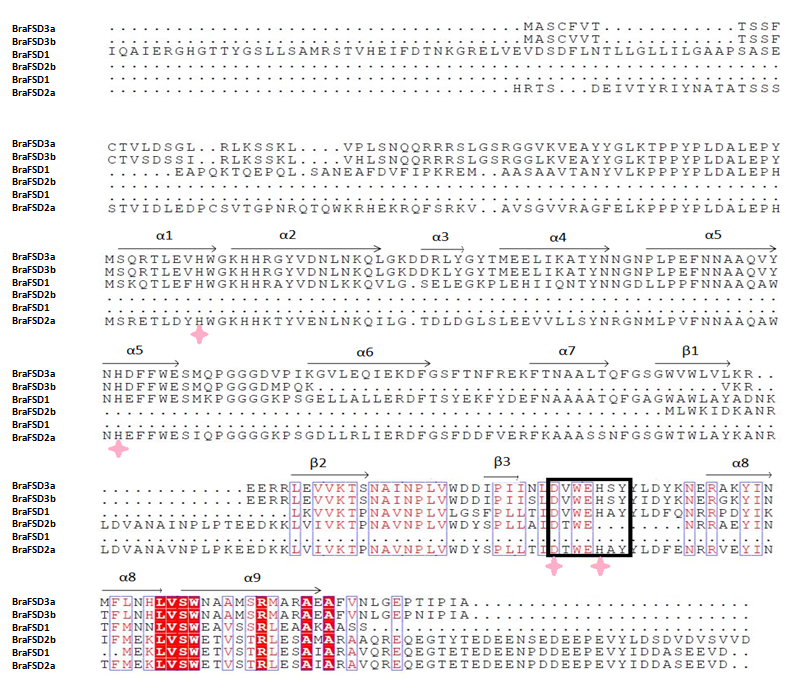

Supplement: Supplementary file 3 — a Figure depicts the multiple sequence alignment of Cu-ZnSODs from B. rapa, anti-parallel β-strands are shown by over head red arrows, Cu binding residues are shown in yellow asterisk, Zn residues in blue asterisk, Bridging histidine is represented by two asterisk. Also various important structural elements are shown like V-loop (Variable loop with over head yellow line), Electrostatic loop (Green) and Greek key loops 1 and 2 (GK1 and GK2 in purple). The Cu2+ and Zn2+ motif signature sequence is highlighted in black box. b Figure shows the multiple sequence alignment of MnSODs from B. rapa, wherein their metal binding sites is highlighted in black box and the metal binding residues are marked with blue asterisks. Also the α-helices and β-sheets are shown by over head black arrows. c Figure shows the multiple sequence alignment of FeSODs from B. rapa, wherein their metal binding sites is highlighted in black box and the metal binding residues are marked with pink asterisks. Also the α-helices and β-sheets are shown by over head black arrows. (ZIP 2377 kb) [file 12864_2019_5593_MOESM3_ESM.zip › Additional file 7c.tif]

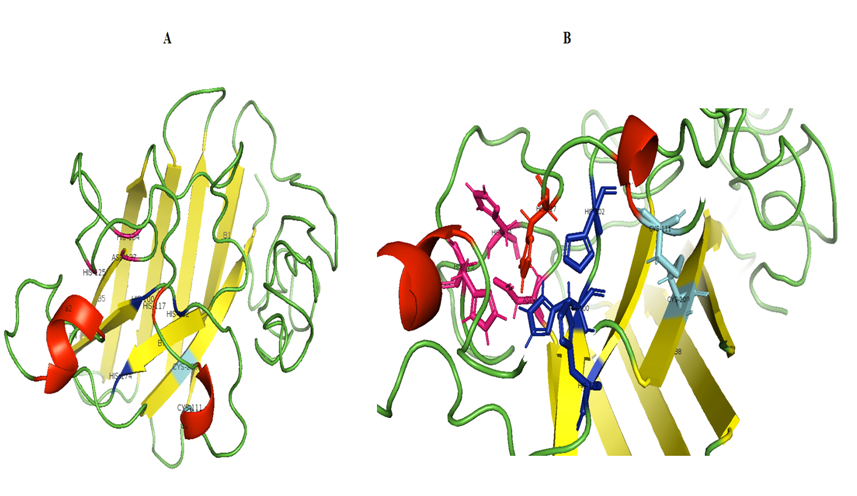

Supplement: Supplementary file 4 — Three dimensional structure of Cu-ZnSOD determined using PyMOL shows the occurrence of eight anti-parallel β-sheets and two α-helices (A). Cu2+ and Zn2+ binding residues are shown in blue and pink colored sticks with the bridging histidine highlighted with red stick and the disulphide bond formed by two Cys-Cys is shown in cyan color (B). (TIF 347 kb) [file 12864_2019_5593_MOESM4_ESM.tif]

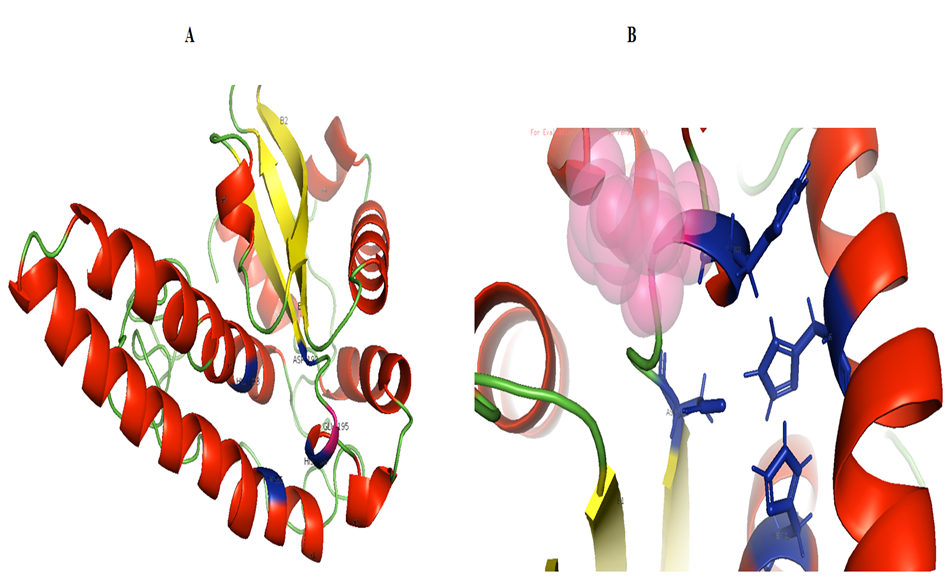

Supplement: Supplementary file 5 — Three dimensional structure of MnSOD determined using PyMOL shows the occurrence of three anti-parallel β-sheets and seven α-helices (A). The metal binding residues are shown in blue sticks which form a bipyramidal structure in the presence of H2O. Also the conserved Glu residue is marked with pink sphere (B). (TIF 539 kb) [file 12864_2019_5593_MOESM5_ESM.tif]

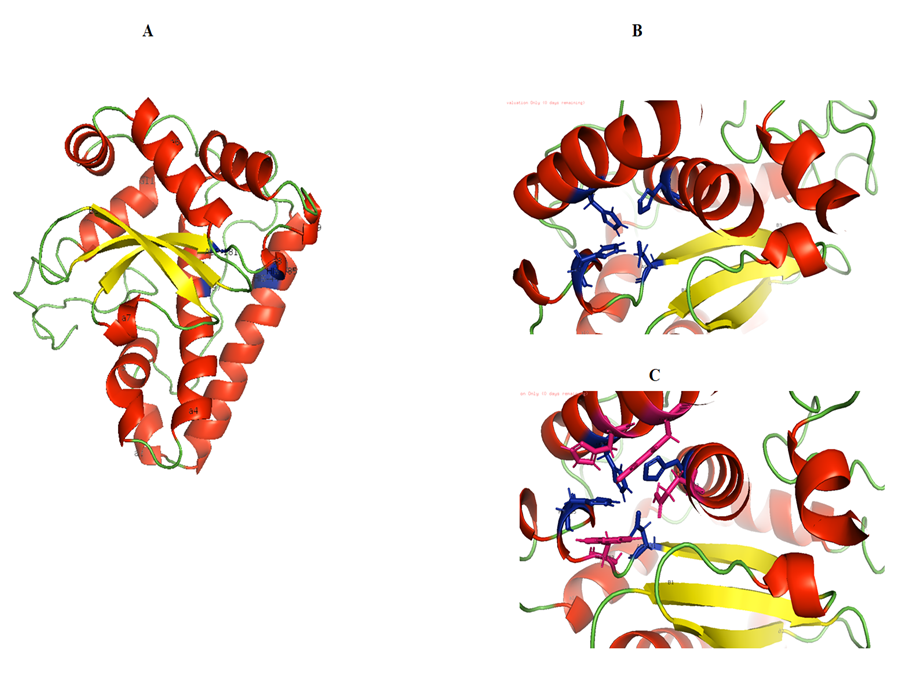

Supplement: Supplementary file 6 — Three dimensional structure of FeSOD determined using PyMOL shows the occurrence of three anti-parallel β-sheets and nine α-helices (A). The metal binding residues are shown in blue sticks which form a bipyramidal structure in the presence of H2O (B). Also the shell forming residues are highlighted with pink sticks (C). (TIF 457 kb) [file 12864_2019_5593_MOESM6_ESM.tif]

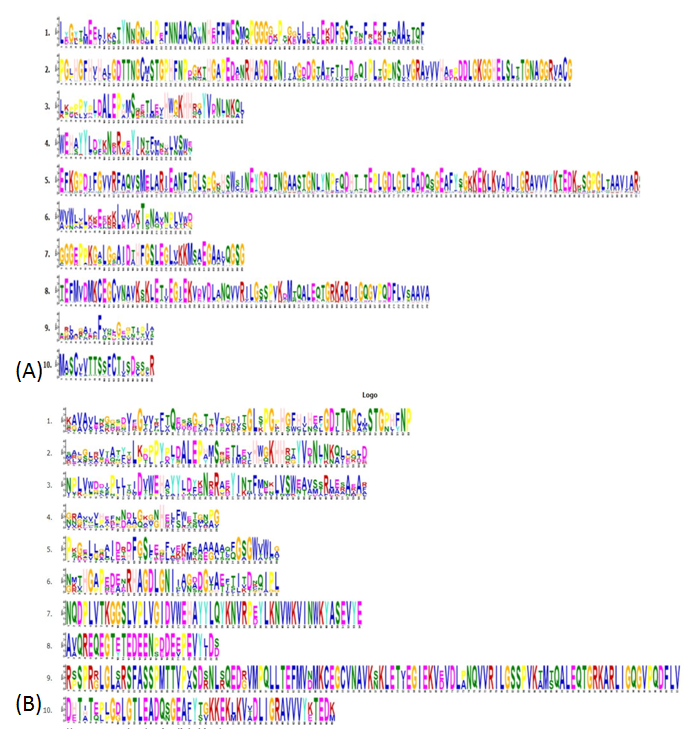

Supplement: Supplementary file 8 — Conserved motif logos in (a) BjuSOD and (b) BraSOD proteins. (TIF 530 kb) [file 12864_2019_5593_MOESM8_ESM.tif]

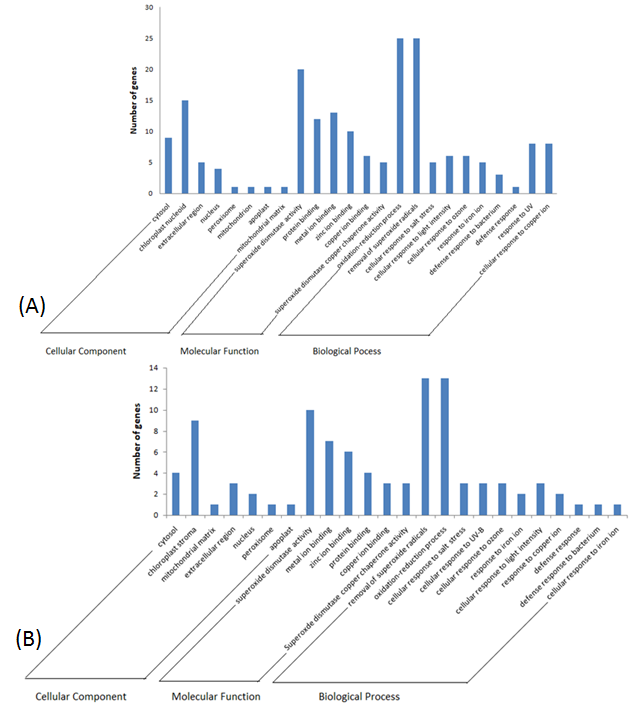

Supplement: Supplementary file 9 — Functional classification of (a) BjuSOD and (b) BraSOD genes on the basis of Gene Ontology (GO) terms assigned to various genes using BLAST2GO tool. GO terms enrichments in 3 different categories i.e. i) Cellular Component, ii) Molecular Function and iii) Biological Process were predicted. (TIF 126 kb) [file 12864_2019_5593_MOESM9_ESM.tif]

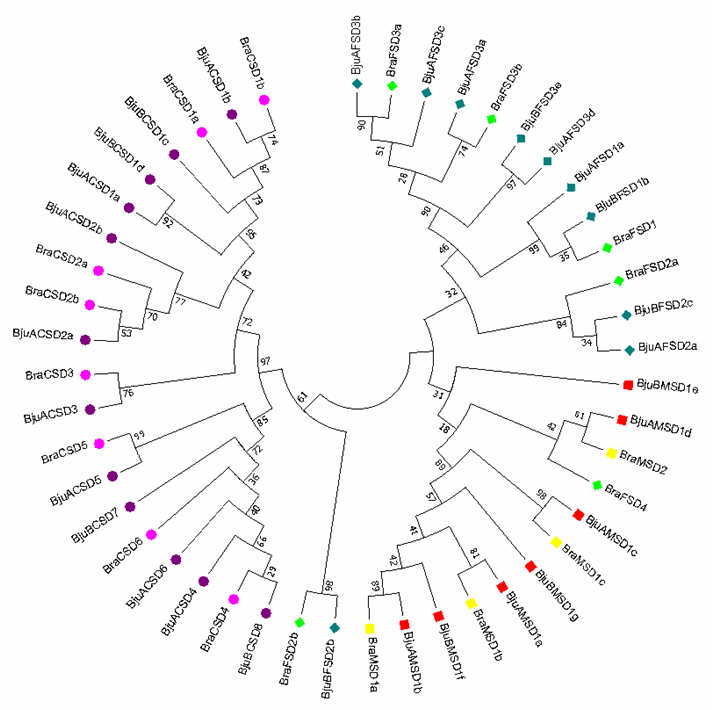

Supplement: Supplementary file 10 — Phyolgenetic tree of SOD genes from B. rapa and B. juncea. Neighbor-Joining analysis was performed with a bootstrap value of 1000 using Mega 7 program. Poisson correction method was used to compute the evolutionary distance. (TIF 141 kb) [file 12864_2019_5593_MOESM10_ESM.tif]

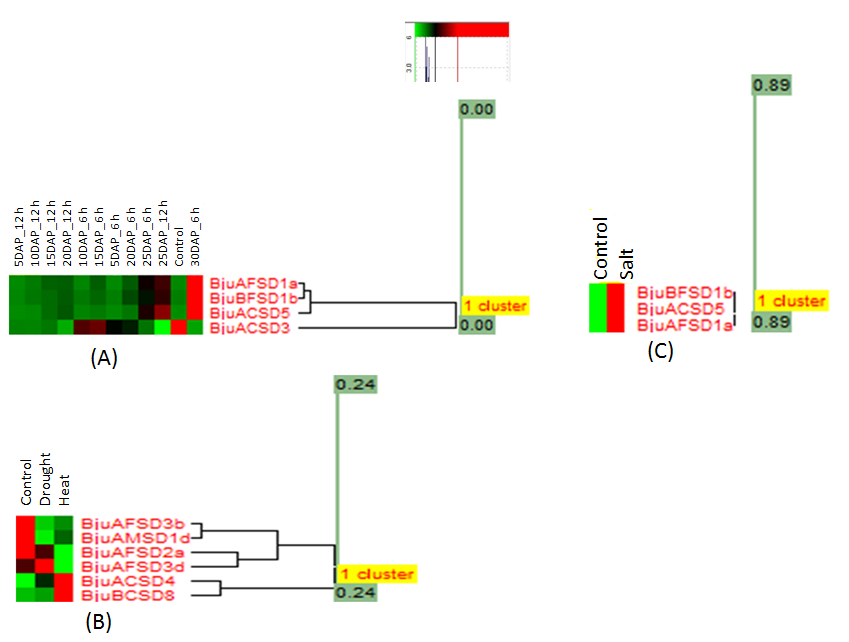

Supplement: Supplementary file 11 — Expression analysis of BjuSOD genes under abiotic stresses. The differential expression profile is shown by Heat map under cold stress (A), Heat and drought stress (B), Salt stress (C). The HCE3.5 software was used to cluster together the genes showing similar expression pattern. (TIF 139 kb) [file 12864_2019_5593_MOESM11_ESM.tif]

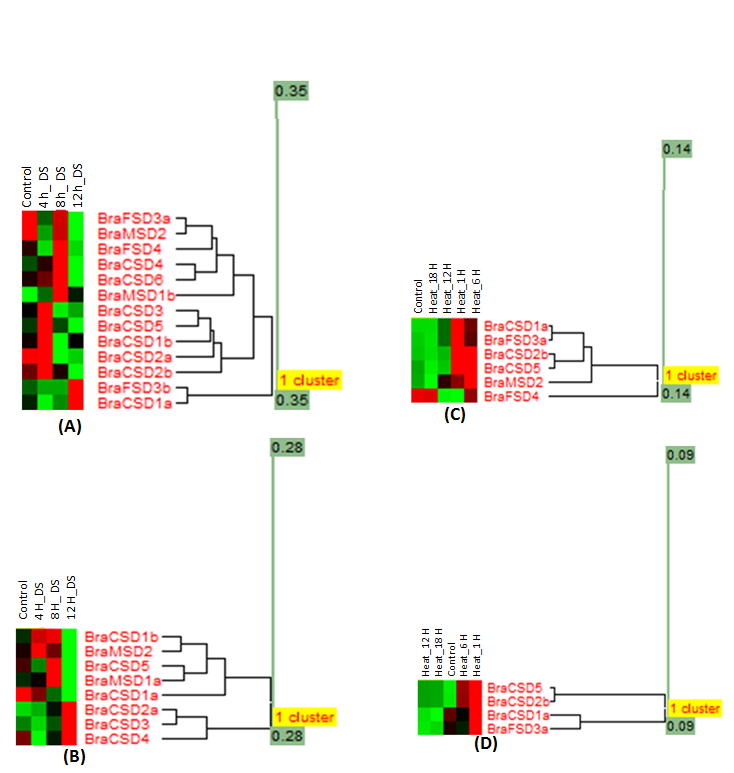

Supplement: Supplementary file 12 — Expression analysis of BraSOD genes under abiotic stresses. The differential expression profile is shown by Heat m ap under drought stress (DS) in drought sensitive variety (A) and drought tolerant variety (B); Heat stress in Heat sensitive variety (C) and Heat tolerant variety (D). The HCE3.5 software was used to cluster together the genes showing similar expression pattern. (TIF 190 kb) [file 12864_2019_5593_MOESM12_ESM.tif]
