# Supplementary material for: Genome-wide identification and characterization of abiotic-stress responsive SOD (superoxide dismutase) gene family in Brassica juncea and B. rapa
Source: BMC Genomics. 2019 Mar 19;20:227. doi: 10.1186/s12864-019-5593-5 (PMC6425617; doi:10.1186/s12864-019-5593-5)
Supplement: Supplementary file 7 — Motif sequences of (a) BjuSOD and (b) BraSOD proteins identitifed by MEME tool. (DOCX 14 kb) [file 12864_2019_5593_MOESM7_ESM.docx]

**Additional file 2**

**(a) Motif sequences of BjuSOD proteins identitifed by MEME tool.**

| Motif Number | Width | Protein Sequence | pfam domain |
| --- | --- | --- | --- |
| 1 | 77 | LYGYTLEELIKATYNNGNPLPEFNNAAQAYNHEFFWESMQPGGGGKPSKGLLELIEKDFGSFTNFREKFTNAALTQF | IMC |
| 2 | 108 | PGLHGFHVHALGDTTNGCMSTGPHFNPDGKTHGAPEDANRHAGDLGNITVGDDGTATFTITDSQIPLTGPNSIVGRAVVVHAEPDDLGKGGHELSLTTGNAGGRVACG | SOD_Cu |
| 3 | 39 | LKPPPYPLDALEPYMSQETLEVHWGKHHRGYVDNLNKQL | IMA |
| 4 | 28 | DVWEHAYYLDYKNRRPEYINTFMNHLVSWE | IMC |
| 5 | 150 | EFKGPDIFGVVRFAQVSMELARIEANFTGLSPGKHSWSINEYGDLTNGAASTGNLYNPFQDHTNTEPLGDLGTLEADQSGEAFYSGKKEKLKVADLIGRAVVVYKTEDKKSGPGLTAAVIARSAGVGENYKKLCTCDGTVIWEATDSDFV | SOD_Cu |
| 6 | 28 | WVWLVLKKEEKKLVVVKTPNAVNPLVWD | IMC |
| 7 | 78 | TEFMVDMKCEGCVNAVKSKLETIEGIEKVDVDLANQVVRILGSSPVKDMTQALEQTGRKARLIGQGVPQDFLVSAAVA | HMA |
| 8 | 39 | GGGEPPKGALGGAIDTHFGSLEGLVKKMSAEGAALQGSG | IMC |
| 9 | 20 | SRLAGAIAFVQLGEGTIPIA | None |
| 10 | 20 | MASCVVTTSSFCTISDSSSR | IMA |

**(b) Motif sequences of BraSOD proteins identitifed by MEME tool.**

| Motif number | Width | Protein Sequence | pfam domain |
| --- | --- | --- | --- |
| 1 | 63 | KAVAVLKGGEDVEGVVRFTQESSGPTTVTGRISGLSPGPHGFHIHEFGDTTNGCISTGPHFNP | CZ domain |
| 2 | 55 | SALGLKVTATYTLKPPPYPLDALEPHMSRETLEIHWGKHHRAYVDNLNKQLEGLD | IMA domain |
| 3 | 55 | NPLVWDDIPLJTIDVWEHAYYLDYKNRRAEYINTFMNKLVSWEAVSSRLESAEAF | IMC domain |
| 4 | 28 | GRAVVVPEFNBDLGKGNHELFWETGNPG | No match |
| 5 | 39 | PKGELLGAIDRDFGSLEGFVEKFSAAAAAQFGSGWVWLA | IMC domain |
| 6 | 39 | NKTHGAPEDENRHAGDLGNIIAGADGVAEFTITDNQIPL | CZ domain |
| 7 | 54 | NQDPLVTKGGSLVPLVGIDVWEHAYYLQYKNVRPEYLKNVWKVINWKYASEVYE | IMC domain |
| 8 | 28 | AVQREQEGTETEDEENPDDEEPEVYJDD | No match |
| 9 | 112 | RPSPRGLGLARSFASSPMTTVPVSDRNLRQEDGVMPQLLTEFMVDMKCEGCVNAVKNKLETIEGIEKVEVDLPNQVVRILGSSPVKAMSQALEQTGRKARLIGQGVPQDFLV | HMA domain |
| 10 | 49 | DHTATEPLGDLGTLEADQSGEAFYTGKKEKLKVVDLIGRAVVVYKTEDK | CZ domain |
